# Cross-dimension serial dependence effects between numerosity and duration

**DOI:** 10.1186/s12915-026-02701-9

**Published:** 2026-07-27

**Authors:** Michele Fornaciai, Irene Togoli, Olivier Collignon

**Affiliations:** 1 Psychological Sciences Research Institute (IPSY) And Institute of Neuroscience (IoNS), Université Catholique de Louvain, Louvain-La-Neuve, Belgium; 2 Life Sciences Department, University of Trieste, Trieste, Italy

**Keywords:** Serial dependence, Magnitude integration, Numerosity perception, Time perception

## Abstract

**Background:**

Our perception and decisions are not only driven by present information, but are also influenced by past information. For instance, previous stimuli can affect the judgement of current ones in an attractive way—a phenomenon known as “serial dependence.” Serial dependence has been shown to occur even across stimuli with different low-level features, suggesting the involvement of high-level computations. Here we further address the level of abstraction at which serial dependence originates by investigating biases across different perceptual dimensions, that is, numerosity and duration—two “magnitude” dimensions that usually bias each other when modulated together. Participants were shown sequences of briefly presented dot-arrays, and asked to reproduce either their duration or the average numerosity computed across the sequence, in separate conditions. We then assessed the influence of duration on numerical estimates, and vice versa, both within the same stimulus (“magnitude integration”) and across successive stimuli (“serial dependence”).

**Results:**

Our results show significant influences across the two dimensions, occurring both within the same stimulus and across successive stimuli. Moreover, we show that the strength of serial dependence can be predicted based on the strength of magnitude integration, suggesting a relationship between the two effects.

**Conclusions:**

Our findings demonstrate that serial dependence can occur between two different perceptual dimensions when they provide compatible information (i.e., “more” vs. “less”), and thus that serial dependence originates from computations shared across different magnitude dimensions. The relationship between serial dependence and magnitude integration additionally suggests a reliance on similar mechanisms, integrating both past and present magnitude information to build a generalized estimate of magnitude.

**Supplementary Information:**

The online version contains supplementary material available at https://doi.org/10.1186/s12915-026-02701-9.

## Background

What we see in the present—and how we judge it—is not only determined by the information reaching our senses at any given time, but it also depends on past information, that is, our perception and decisions in the recent past. The context in which sensory inputs are embedded plays indeed an important role in determining how they are converted into a subjective percept. For example, after a long, sustained exposure to a specific stimulus, what we see after is strongly distorted in a *repulsive* fashion, that is, away from the “adaptor” stimulus. This adaptation process leads indeed to robust perceptual aftereffects and biases (e.g., see for instance [1] for a review). While adaptation usually requires a relatively long exposure to a stimulus, biases across successive stimuli can be observed even with much shorter presentations. For instance, in a sequence of brief stimuli, our perception or judgement of a present stimulus can be *attracted* towards its preceding stimulus, so that the current stimulus is judged to be more similar to the preceding one than it actually is in reality—a phenomenon called “serial dependence” [2–6].

Serial dependence effects have been shown to emerge in virtually every dimension of perceptual experience, including both fundamental attributes such as orientation [5], color [7], numerosity [2, 8], and motion [9], as well as more complex features such as face identity [10] or emotions [11]. Even if it has been mostly investigated in vision, serial dependence also arises in audition [12]. This suggests a generalized role of serial dependence in defining our perceptual experience, not limited to a specific dimension, although the properties of the effect often differ in different domains and tasks [8, 13–15].

The fact that serial dependence abstracts from the low-level sensory features of the stimuli supports a process originating beyond the primary sensory stages of information processing. For instance, in orientation perception, the effect occurs even if successive stimuli have different colors [16]. In numerosity perception, the effect occurs even if two successive stimuli are presented in different formats: the numerosity of a sequence of brief events presented over time can affect the perceived numerosity of an array of items presented in space [17, 18]. The effect has also been shown to occur across different sensory modalities (vision and audition), although considering a dimension that by itself is very abstract like the subjective rating of emotional valence and arousal triggered by a stimulus [11]. These results suggest that serial dependence originates from a stage at which a relevant property of the stimulus has been already extracted and represented in at least a partially abstract fashion, separately from its low-level sensory properties.

This property of serial dependence raises the interesting possibility that the bias might occur even across two different perceptual dimensions, provided that different types of information are represented in a compatible form. This might be the case, for instance, for the magnitude dimension of visual stimuli, such as numerosity and duration. Magnitude dimensions are indeed intrinsically linked [19] and can interact when modulated together [20–22]. The “magnitude integration” effect can manifest by shifting the perception of a magnitude toward another “interfering” dimension. For example, the numerosity of an array of items presented for a long time is perceived to be higher than the same numerosity presented for a shorter time, and vice versa, the duration of a numerous set of items seems longer compared to a less numerous set [21–24]. The magnitude integration effect is however not always symmetrical, but sometimes it appears to be stronger in one direction compared to the other. Duration is usually the most susceptible dimension, being easier to bias by other dimensions such as size or numerosity, and exerting weaker effects [25]. This is especially true when using static stimuli (e.g., an individual array of dots presented for a given amount of time; [22]). Instead, we have recently shown that magnitude integration between numerosity and duration becomes symmetrical when using dynamic stimuli that make the non-temporal dimension to unfold over time [22, 24]. This is likely due to the processing time-course of different magnitudes. Namely, the numerosity of a static array is processed much faster than duration, which needs the entire interval to unfold before it could be processed. This would thus make numerosity more resistant to biases, since by the time duration is processed numerosity has been already represented in a stable fashion. Finally, cross-dimension interactions can also occur via perceptual adaptation [26, 27]. Duration adaptation can indeed affect numerical estimates, although numerosity adaptation has only a weak effect on duration [27]. Both these dimensions are also similarly affected by motion adaptation, suggesting partially shared processing mechanisms [28, 29].

Considering these properties of magnitude perception, we asked whether serial dependence originates from a processing level high enough to involve “abstract” magnitude information. Can serial dependence work across two different magnitude dimensions? Understanding the level of abstraction at which serial dependence originates would provide new insights into the mechanisms of this phenomenon, and its relationship with other perceptual effects such as magnitude integration. Considering the relationship between different magnitudes and the high-level properties of the serial dependence effect, in principle cross-dimension serial effects should be possible. Previous results addressing this question did not show mutual serial dependence effects across different magnitudes, but only a repulsive influence of duration on numerosity [30]. However, this study employed static stimuli that we now know are not optimal to engage the shared mechanisms of magnitude processing due to the imbalance in the processing time-course [22], leaving the question open.

In the present study we address the existence of cross-dimension serial dependence effects between numerosity and duration by using dynamic stimuli, and a paradigm optimized to induce magnitude integration effects. In two conditions performed by the same participants, we presented dynamic sequences of briefly-presented dot-arrays (50 ms each; 20 Hz presentation frequency) and asked the participants to judge either the average numerosity of the sequence or its duration. With this setup, even a non-temporal dimension such as numerosity requires processing over time to compute the average of the sequence, which facilitates a link with duration [22, 31]. While judging one dimension, the other irrelevant magnitude was modulated, alternating different ranges in different blocks of trials to modulate the strength of the effect. Namely, we alternated two ranges of the irrelevant magnitude so that the higher portion of the first range becomes the lower portion of the second range, inducing a recalibration that may modulate the strength and/or direction of the effect. Due to the change in the stimulation range and the overlap between the upper and lower portion of the two ranges, we predict at least a reduced effect in the second range. We then assessed the magnitude integration bias within the same stimulus, and serial dependence effects across successive stimuli, as well as the relationship between the two biases. Our hypothesis is that, mirroring magnitude integration, past durations should affect current numerical estimates in an attractive fashion, and vice versa, past numerosities should affect current duration estimates. If so, modulating the extent to which different magnitudes influence each other by using different ranges should also proportionally affect serial dependence effects across the two dimensions. Finally, since the two phenomena potentially hinge upon the same information integrated within the same stimulus representation or across different representations, their strength should be linked – that is, the stronger the magnitude integration effect, the stronger the serial dependence effect.

## Results

In this study, we addressed the existence of serial dependence effects across different magnitude dimensions to gain further insights into the level of abstraction at which serial dependence originates. Perceptual dimensions such as numerosity and duration are indeed intrinsically linked as magnitudes and usually interact, biasing each other when modulated together. Due to this link, our hypothesis is that the duration of a previous stimulus should affect the perceived numerosity of a current stimulus, and vice versa. Moreover, if cross-magnitude serial dependence works based on information originating at a similar stage compared to magnitude integration, then the two phenomena might be linked.

**Fig. 1:**
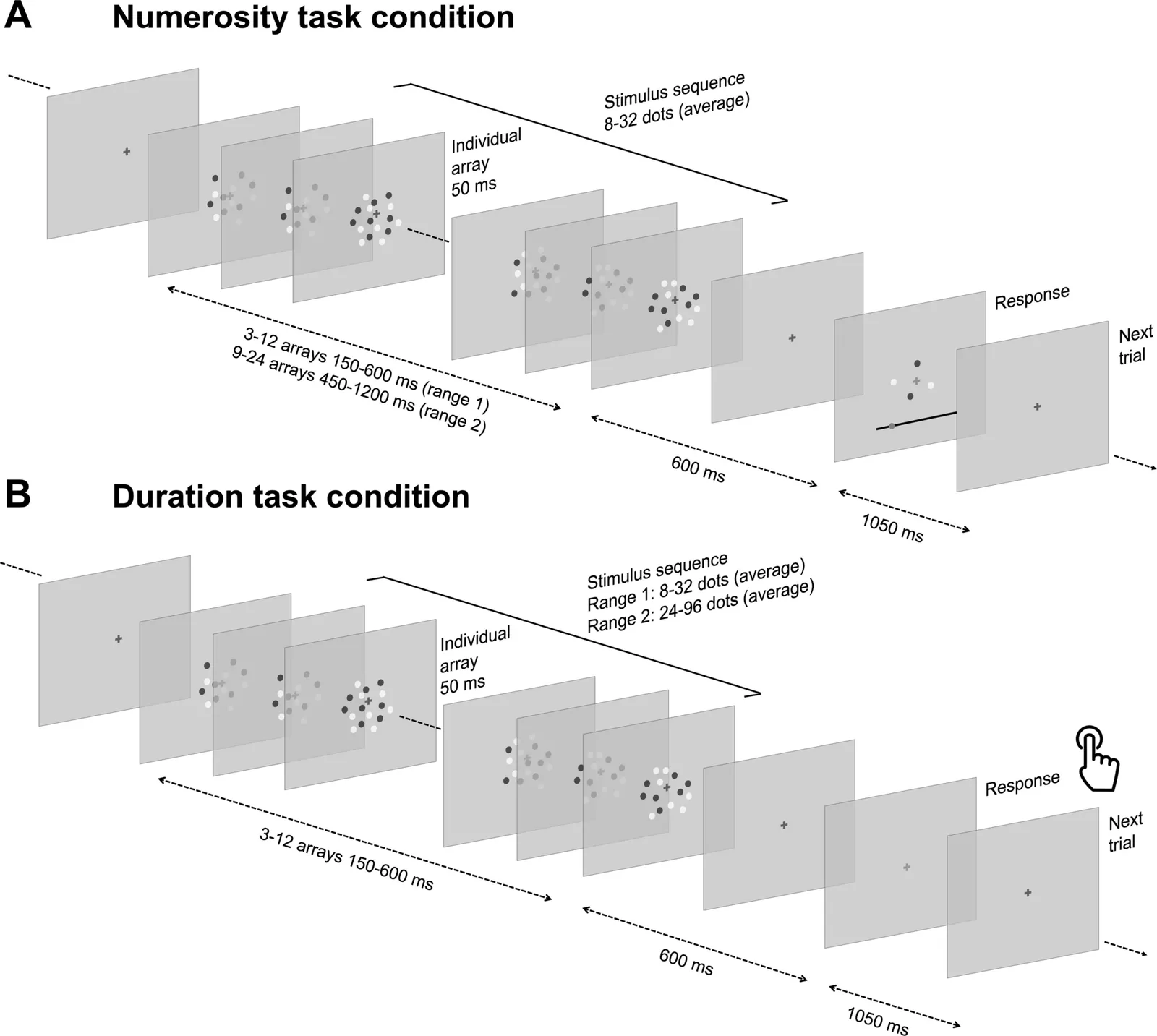
Experimental procedure.** A** Numerosity task condition. In the numerosity task condition, participants were instructed to focus on the numerosity of the sequence presented in each trial, and to judge the average numerosity presented across the different arrays. While participants kept their gaze on the central fixation cross, the stimulus sequence was presented at the center of the screen, for a variable amount of time (duration range 1 = 150–600* ms*, used in blocks 1, 3, and 5; duration range 2 = 450–1200* ms*, used in blocks 2 and 4). The average numerosity of each sequence (i.e., the task-relevant dimension) ranged from 8 to 32 dots, with the numerosity of each individual array varying ± 50% around the selected average. After the offset of the sequence, a blank interval of 600 ms preceded the response phase. In the response phase, a slider appeared at the bottom of the screen, and participants were instructed to move the cursor to reproduce the average numerosity of the sequence. Moving the cursor from left to right made dots to progressively appear at pseudo-random positions around the center of the screen (response range = 1–40 dots). Participants could move the cursor back and forth to adjust the array with no time limitations. Once the response array was judged to match the perceived average numerosity of the sequence, a click of the mouse button confirmed the response and started the next trial after an interval of 1050 ms. **B** Duration task condition. The stimulation procedure in this task was similar to the numerosity task. However, here the duration of the sequence ranged from 150 to 600 ms in all blocks. The average numerosity was instead varied according to two different ranges, spanning 8–32 dots in block 1, 3, and 5 (numerosity range 1) and 24–96 in block 2 and 4 (numerosity range 2). In the response phase of the duration task, participants were asked to press and hold the space bar of a standard computer keyboard to reproduce the duration of the sequence. The stimuli are not depicted in scale

In the study, the participants (*N* = 28) performed a numerosity and a duration reproduction task of dynamic stimuli (i.e., fast sequences of dot arrays), in which both numerosity and duration were concurrently modulated (Fig. 1). Each stimulus sequence was composed by a series of briefly-flashed individual arrays, presented at 20 Hz (i.e., 50 ms each). This design was chosen to give the impression of a fast dynamic modulation of numerosity rather than a sequence of static arrays, in line with the design used in previous studies [22, 31]. Note that in this design the duration of the stimuli corresponds to the number of individual arrays presented. For consistency, henceforth we will describe the stimuli in terms of their duration. While in each task the range of the task-relevant magnitude was kept constant throughout the 5 blocks composing the sessions (8–32 dots and 150–600 ms, respectively for the numerosity and the duration task), the range of the task-irrelevant, interfering dimension was alternated. This was done to introduce a potential modulation of the magnitude integration effect (i.e., a reduction in the effect due to the overlap between upper and lower portion of the two ranges, which requires a re-calibration of the magnitude mapping), and to assess the flexibility of the effects in face of changes in the stimulation statistics. Namely, in the numerosity task (Fig. 1A), the duration of the stimuli was 150–600 ms in block 1, 3, and 5, while it was 450–1200 ms in block 2 and 4. In the duration task (Fig. 1B), the average numerosity of the stimuli was 8–32 dots in block 1, 3, and 5, and 24–96 dots in blocks 2 and 4. In order to increase the efficiency of the experiment and facilitate data analysis, the order of the two ranges was kept fixed across participants. Although a counterbalanced presentation would be elegant, it would have required to test two independent samples to avoid averaging together effects induced by different magnitude levels. Indeed, a counterbalanced order within the same group of participants would have meant averaging together the effects induced by different magnitudes. For instance, the effect of a “low” magnitude would be the average of, for instance, 8 dots and 24 dots. This in turn would have introduced a confounding factor in our quantitative, parametric modulation of magnitude. The different ranges used have been chosen to be equally effective, in principle, in inducing magnitude integration and serial dependence effects so it is unlikely that this methodological choice had an impact on our main findings (see Discussion for more information). Besides this argument, the partial overlap between different ranges provides a way to assess whether any modulation of the effect we might observe across blocks is due to the order of presentation or the specific magnitudes presented. Regarding the use of these two magnitude ranges in different blocks, our hypothesis concerns the flexibility of the magnitude processing system in face of changes in the stimulation statistics. Specifically, once the magnitude processing system gets calibrated to a specific range of the interfering magnitude at the beginning of the session, it may not be flexible enough to immediately recalibrate the effect when facing a different range. Indeed, since the higher end of the first range corresponds to the lower end of the second range, a complete recalibration is needed to keep the effect relative to the current stimulation statistics. By changing the range of the interfering magnitude, we thus aimed at assessing such recalibration. According to the idea of a link between magnitude integration and serial dependence effects, the change of range should similarly modulate both effects, providing an additional way to test for their relationship. In our analysis, we assessed the biases induced by duration on numerosity and by numerosity on duration as a function of these different ranges, both within the same stimulus (magnitude integration) and across successive stimuli (serial dependence).

**Fig. 2:**
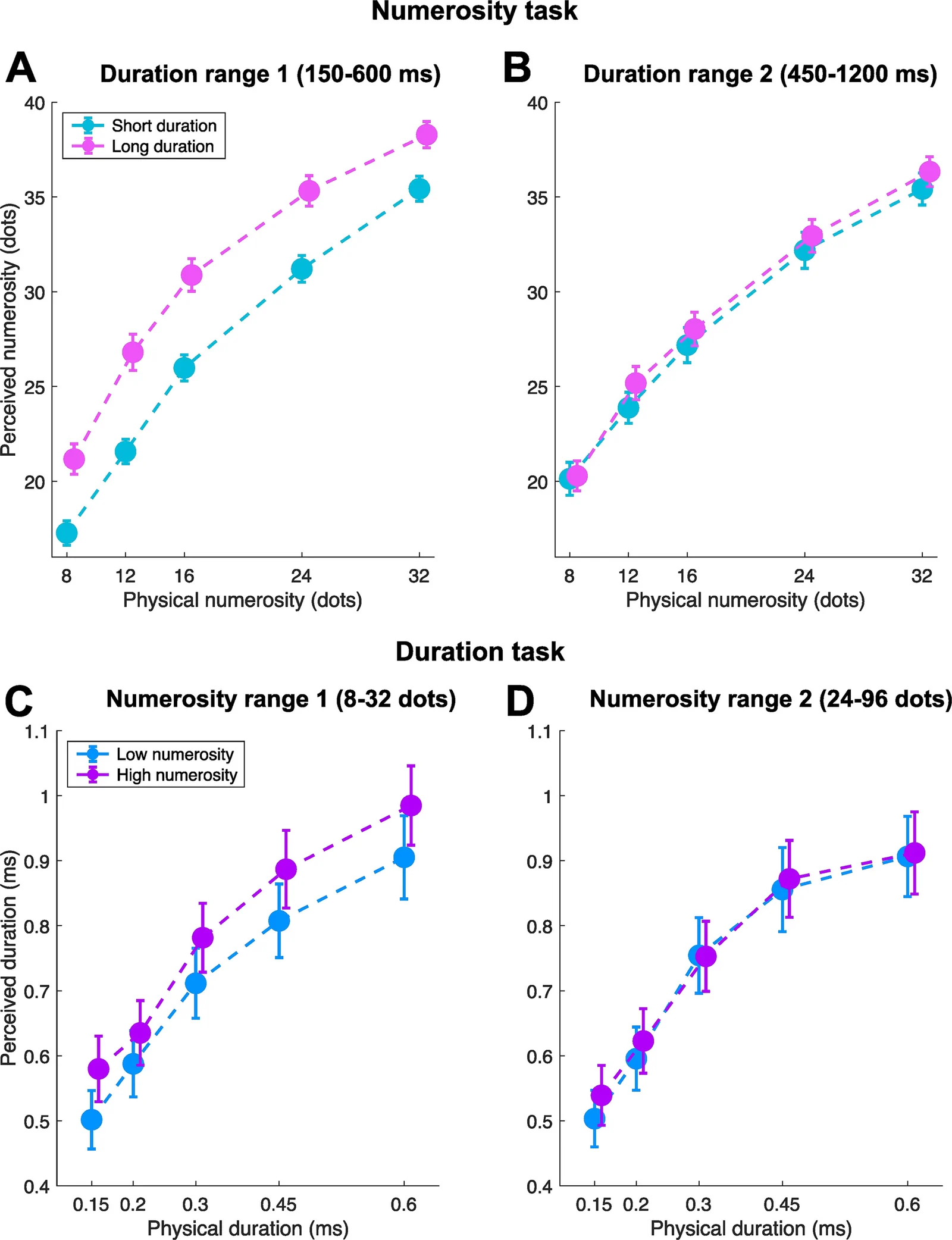
Magnitude integration effects.** A** Average numerical estimates corresponding to the different levels of average numerosity in the numerosity task. The data is plotted separately according to the duration of the stimuli (duration range 1). Namely, according to whether the duration was shorter (15–200* ms*; cyan data points) or longer (450–600 ms; magenta data points) compared to the middle value of the range. **B **Average numerical estimates in block 2 and 4 (duration range 2), plotted separately for shorter (450–600* ms*) and longer (950–1200* ms*) durations. **C** Average duration reproduction responses plotted separately for low and high numerosities (range 1). **D** Average duration reproduction responses in block 2 and 4 (range 2). Error bars are SEM

First, we assessed the magnitude integration effects across numerosity and duration. Since we alternated different ranges of the task-irrelevant dimension in different blocks, we analyzed the effect separately for blocks 1, 3, 5 (range 1) and 2, 4 (range 2). To assess the general level of performance, we computed the precision in the task in terms of Weber’s fraction (i.e., standard deviation of responses divided by the average response; WF). In the numerosity task, responses were overall quite precise, both when numerosity was coupled with the first (average WF ± SD = 0.18 ± 0.07) and the second duration range (average WF ± SD = 0.19 ± 0.07). In the duration task, precision was, unsurprisingly, lower, which is in line with the higher difficulty of judging visual durations as opposed to other visual dimensions [32]. When duration was coupled with the numerosity range 1, the average WF was 0.30 ± 0.11. When coupled with the numerosity range 2, the average WF was 0.33 ± 0.14. The relationship between the performance in the two tasks was assessed with a linear mixed-effect (LME) on the average WFs across the group. The model included the WF in the numerosity task as dependent variable, the WFs in the duration task and the range as fixed effects, and the subjects as the random effect. Performance in the two tasks did not show a relationship (model adj. *R*^2^ = 0.91; *p* = 0.86). No effect of range (*p* = 0.31) or interaction between the two factors (*p* = 0.24) was observed as well. This suggests that the two tasks involve at least partially different response mechanisms. In terms of accuracy, we observed large, general overestimation biases in both task (see Fig. 2). In the numerosity task, the different average numerosities (8, 12, 16, 24, 32 dots) were reproduced as (mean ± SD) 19.72 ± 4.03, 24.36 ± 4.29, 28.02 ± 4.37, 32.91 ± 4.32, and 36.37 ± 3.88 dots. In the duration task, the different durations (150, 200, 300, 450, 600 ms) were reproduced as 531 ± 240 ms, 610 ± 259 ms, 750 ± 285 ms, 855 ± 312 ms, and 927 ± 325 ms. Although we observed robust biases in terms of absolute judgements, what is more important in this context is the relative difference in magnitude estimates based on the interfering dimension (i.e., the difference between cyan and magenta data points plotted in Fig. 2).

When assessing the magnitude integration effect on perceived numerosity, the first duration range used in blocks 1, 3, and 5 was highly effective in inducing a bias (Fig. 2A). When the stimuli had a longer duration (450–600 ms), we observed higher numerical estimates compared to when the numerosities were coupled with shorter durations (150–200 ms). A LME model on the average numerical estimates (adjusted *R*^2^ = 0.88), including both duration and numerosity as fixed effects (plus the subjects, numerosity, and duration as random intercepts), showed a significant effect of numerosity (*b* = 4.92, *t* = 13.68, *p* < 0.001) and, importantly, a significant effect of duration (*b* = 5.17, *t* = 6.82, *p* < 0.001). No interaction was observed (*p* = 0.16). While the effect of numerosity in this context reflects the responses to different levels of average numerosity, the effect of duration reflects the magnitude integration bias. When we switched the duration range in block 2 and 4, however, the effect disappeared (Fig. 2B). A LME model (adj. *R*^2^ = 0.90) indeed showed only a significant effect of numerosity (*b* = 3.79, *t* = 11.64, *p* < 0.001), with no effect of duration (*p* = 0.45) and no interaction (*p* = 0.62). In the duration task, the first numerosity range induced consistent biases on duration reproduction responses (Fig. 2C). A LME model (adj. *R*^2^ = 0.92) showed a significant effect of both duration (*b* = 0.09, *t* = 7.77, *p* < 0.001) and numerosity (*b* = 0.06, *t* = 2.27, *p* < 0.024), and no interaction (*p* = 0.66). Here, the magnitude integration effect is indexed by the significant effect of numerosity on duration estimates. When we switched to the second range (Fig. 2D), again the effect disappeared, and the LME model showed only an effect of duration (*b* = 0.1, *t* = 6.63, *p* < 0.001), with no effect of numerosity (*p* = 0.26) and no interaction (*p* = 0.48).

Additionally, we also assessed the relationship between magnitude integration effects across the two tasks. To do so, we performed a LME test on the magnitude integration effects, similar to the model used to the relationship between WFs. The results (adj. *R*^2^ = 0.45) showed no significant relationship between the effects in the two task (*p* = 0.46). We however observed a significant effect of the range (*b* = − 10.436, *t* = − 3.99, *p* < 0.001) reflecting the reduced bias yielded by the second range, but no interaction between magnitude integration and range (*p* = 0.075).

Finally, since the judgments in the numerosity task have the response time (RT) as an additional factor to take into account, we also assessed the average RTs as a function of the different magnitudes. This served two purposes: (1) to assess whether RTs vary as a function of the magnitude being estimated or as a function of the interfering magnitude, and (2) to control for the potential influence of the compatibility of response codes with the different magnitudes. Indeed, since the response involved a left-to-right slider, associations between the side at which the response occurs and the magnitude can influence the judgment. Typically, lower magnitudes are associated with the left space, and higher magnitude with the right space [33–37]. This in turn could facilitate or hinder the judgment when the magnitude is consistent or inconsistent with the spatial location where the response takes place. Overall, we did not find any significant difference in response times in the numerosity task as a function of numerosity or duration (neither with the duration range 1, nor with the range 2), suggesting that the compatibility of the response codes with relevant and irrelevant magnitudes did not affect the judgments. See the Additional File 1, Fig. S1 [33–40] for more information about this analysis.

**Fig. 3:**
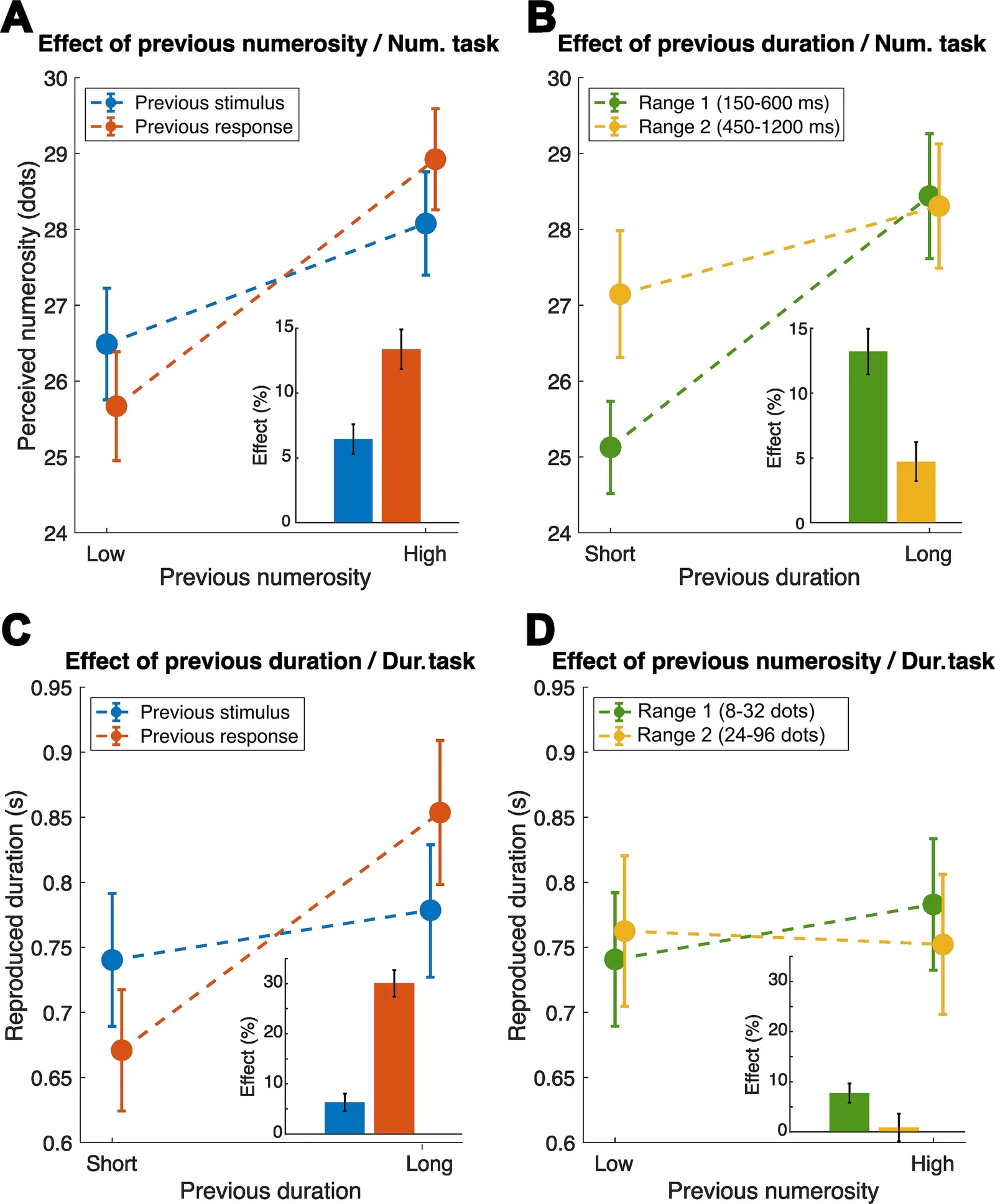
Serial dependence effects.** A** Within-dimension serial dependence effects in the numerosity task. The effect was assessed here as a difference in the average reproduced numerosity of the middle numerosity level (16 dots) as a function of either the previous numerosity (low vs. high compared to the middle numerosity level; blue data points) or the previous response (lower vs. higher than the average response; red data points). The inset on the bottom-right shows the average serial dependence effect in percentage, reflecting the normalized difference between the two data points in the main graph. **B** Cross-dimension serial effects in the numerosity task, plotted separately for the duration range 1 (green data points) and the duration range 2 (yellow data points). The inset shows the average normalized serial dependence effects in percentage. **C** Within-dimension effects in the duration task, computed as a difference in the average reproduced duration of the middle duration level (300 ms) as a function of either the previous duration or the previous response. **D** Cross-dimension effects in the duration task, plotted separately for the two numerosity ranges. Error bars are SEM

Furthermore, we assessed the serial dependence effects both within the same dimension and across different dimensions. To do so, we first focused on the average responses to the stimulus representing the middle level of the two magnitudes (16 dots and 300 ms, respectively for the numerosity and the duration task). When assessing effects within the same dimension, we considered both the stimulus itself and the previous response in two separate analyses, in line with the idea that past responses might provide a better index of serial dependence in reproduction tasks [13, 41]. The serial dependence effects are shown in Fig. 3. In the numerosity task, we observed robust serial dependence effects induced by past numerosities, both in terms of past stimuli and past responses. As shown in Fig. 3A, on average the responses to the middle stimulus level were biased by the preceding numerosity or response in an attractive way. Namely, when the previous stimulus was more numerous or the previous response was higher than average, the current stimulus was over-estimated compared to when the previous stimulus/response was less numerous. The effect of the previous response appears to be noticeably stronger. To better assess the strength of serial dependence, we computed a normalized index reflecting the difference in perceived numerosity of the current stimulus as a function of the previous stimulus or response. This is shown in the inset on the bottom-right of Fig. 3A. A series of *t*-tests showed significant effects both induced by the previous stimulus (average effect ± SD = 6.44% ± 6.22%; one-sample *t*-test against zero: *t*(27) = 5.48, *p* < 0.001, Cohen’s *d* = 1.03) and by the previous response (average effect = 13.38% ± 8.28%; one-sample *t*-test: *t*(27) = 8.55, *p* < 0.001, *d* = 1.62), and that the response induced a significantly stronger effect (paired *t*-test: t(27) = 6.91, *p* < 0.001, *d* = 0.93). More importantly, our results from the numerosity task also showed robust cross-dimension effects, with previous durations affecting current numerical estimates. The effects are significant both when using the duration range 1 (average effect = 13.20% ± 9.52%; one-sample *t*-test: *t*(27) = 7.38, *p* < 0.001, *d* = 1.39) and the duration range 2 (average effect = 4.71% ± 8.08%; one-sample *t*-test: *t*(27) = 3.09, *p* = 0.005, *d* = 0.58), but are significantly stronger in the former case (paired *t*-test: *t*(27) = 3.75, *p* < 0.001, *d* = 0.95).

In the duration task, we observed robust within-dimension serial dependence effects as well (i.e., past durations affecting current duration estimates), again with a predominant influence of past responses (Fig. 3C). Both the effect of past stimuli (average effect = 6.32% ± 9.33%; one-sample *t*-test: *t*(27) = 3.58, p = 0.001, *d* = 0.68) and past responses (average effect = 30.04% ± 14.36%; one-sample *t*-test:* t*(27) = 11.07, *p* < 0.001, *d* = 2.09) were significant, but responses induced significantly stronger effects (paired *t*-test: *t*(27) = 9.58, *p* < 0.001, *d* = 1.88). In terms of cross-dimension effects (Fig. 3D), here the difference in reproduced duration as a function of previous numerosity was less robust than the effect of duration on numerosity. Still, we observed a significant cross-dimension effect when using the numerosity range 1 (average effect = 7.74% ± 10.42%; one-sample *t*-test: *t*(27) = 3.93, *p* < 0.001, *d* = 0.74), although not when using the range 2 (average effect = 0.89% ± 14.93%; one-sample *t*-test: *t*(27) = 0.32, *p* = 0.75, d = 0.06). The effect obtained in the former case was weakly, but significantly higher (paired *t*-test: *t*(27) = 2.07, *p* = 0.048, *d* = 0.18). Overall, these results show that serial dependence occurs across the dimensions of numerosity and duration, and that, similarly to the magnitude integration effect, the bias is reduced when introducing a magnitude range different from the one presented at the beginning of the session.

Do these serial biases genuinely reflect the immediately preceding information, or could they reflect general biases due to the long-term stimulation history? To control for this possibility, we performed an additional analysis. Namely, we focused on the blocks showing the most robust effects (blocks 1, 3, and 5), and assessed the effect by considering the stimulus in a future trial [2, 15], rather than in the past trial (or past mini-block). Specifically, for the within-modal effect, we considered the magnitude (either numerosity or duration) of the corresponding stimulus in the next mini-block. For cross-dimension effects, we considered the magnitude level of the interfering dimension presented throughout the next mini-block. In the numerosity task, the within-dimension effect measured when considering the numerosity of the stimulus in the future trial was (mean ± SD) -0.36% ± 4.90% (one-sample *t*-test,*t*(27) = 0.39, *p* = 0.70). When computing the cross-dimension effect considering the duration in the next mini-block, the effect was 0.91% ± 6.17% (*t*(27) = 0.78, *p* = 0.44). In the duration task, the within-dimension effect of the duration of the future stimulus was 0.07% ± 8.12% (*t*(27) = 0.05, *p* = 0.96), while the cross-dimension effect of the future numerosity was 1.38% ± 9.57% (*t*(27) = 0.76, *p* = 0.45). These results show that the observed serial dependence effects are unlikely to reflect general biases occurring in a longer temporal frame. Note however that using the successive trial or mini-block does not allow to control for very slow drifts in response tendencies or effects occurring at a much broader temporal scale. However, due to the nature of serial dependence and magnitude integration, such potential longer-term biases are not expected to confound the measured effects.

Similarly to the magnitude integration effect, we also assessed whether there is a relationship between serial dependence effects across the numerosity and duration task. First, we used two separate LME models for the within-modal effects, including either the effect of previous stimuli or the effect of previous responses (i.e., effect in the numerosity task as dependent variable and effect in the duration task as fixed effect). In both cases (adj. *R*^2^ = 0.50 and 0.51, respectively), we did not find any significant relationship (*p* = 0.49 and 0.35, respectively). For the cross-dimension effects, we also included the range. The results only showed an effect of range (*b* = − 7.990, *t* = − 3.26, *p* = 0.002), and neither an effect of the serial dependence effect in one task in predicting the other (*p* = 0.77), nor an interaction (*p* = 0.28).

**Fig. 4:**
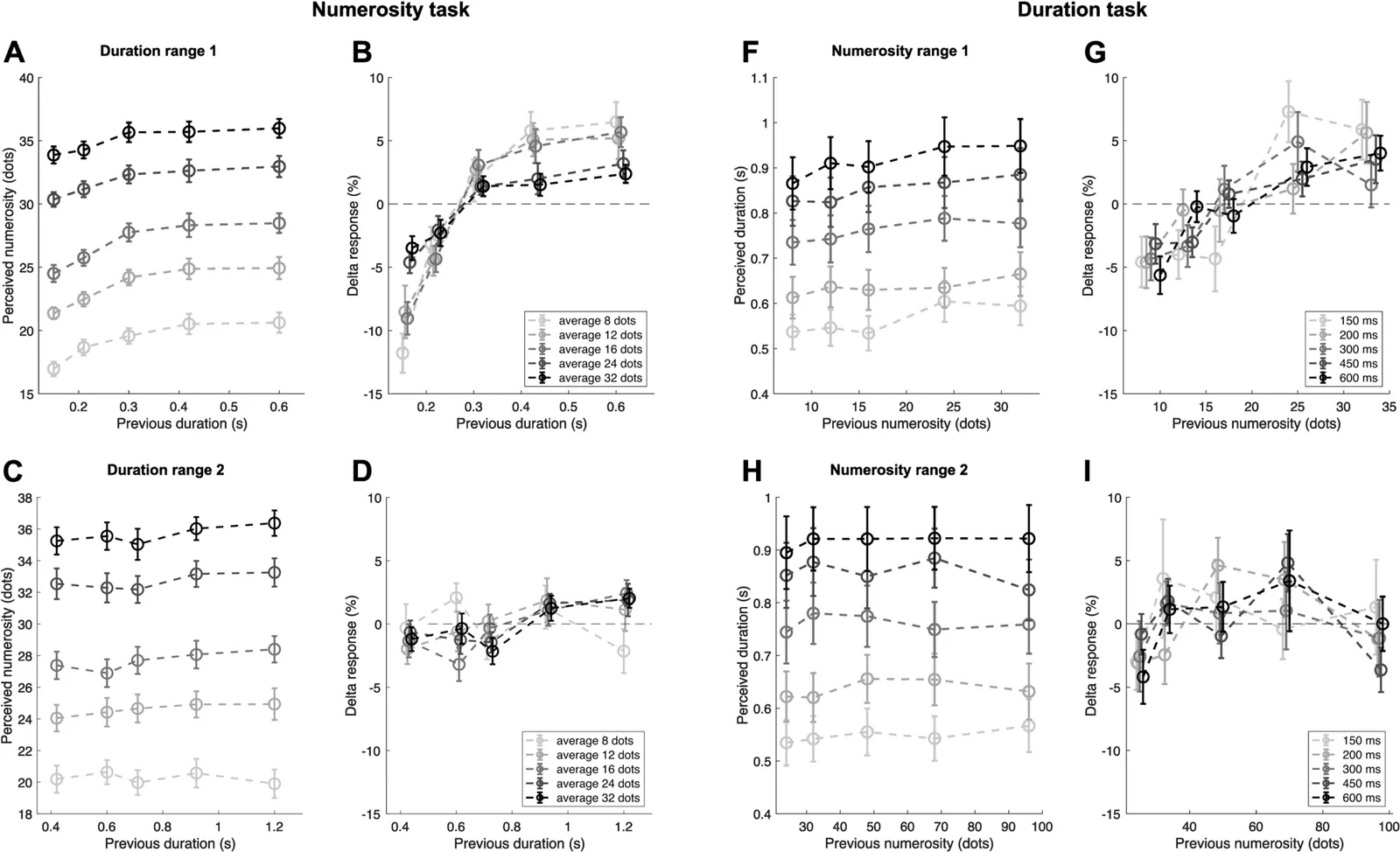
Cross-dimension serial dependence across different combinations of numerosity and duration.** A**–**D** Data from the numerosity task condition. **F**–**I** Data from the duration task condition. **A** Average numerical estimates corresponding to each numerosity as a function of each level of duration (range 1) of the past stimulus. **B** Normalized “delta response” measures corresponding to numerical estimates at each numerosity level as a function of each level of duration (range 1) of the past stimulus. **C** Average numerical estimates for each numerosity level as a function of each level of previous duration (range 2). **D** Delta response corresponding to numerical estimates at each numerosity level as a function of each level of previous duration (range 2). **F** Average duration estimates corresponding to each duration as a function of each level of numerosity (range 1) of the past stimulus. **G** Normalized delta response corresponding to duration estimates at each duration level as a function of each level of numerosity (range 1) of the past stimulus. **H** Average duration estimates for each duration level as a function of each level of previous numerosity (range 2). **I** Delta response corresponding to duration estimates at each duration level as a function of each level of previous numerosity (range 2). Error bars are SEM

To get a more comprehensive account of cross-dimension serial dependence effects, we further assessed the mutual biases across the two dimensions by considering each individual combination of past and present magnitude. Doing so, we thus assessed whether for instance some levels of one magnitude dimension are more prone to biases or more resistant to them [2, 15]. Figure 4 shows the average responses for each level of the task relevant magnitude as a function of each level of the task-irrelevant magnitude in the previous trial (panels A and C for the numerosity task; panel F and H for the duration task). Additionally, we computed a measure of “delta response” based on the normalized difference between responses at each combination of task-relevant and irrelevant dimension and the average of responses within the specific level of the relevant dimension. These measures are shown in Fig. 4B and D (numerosity task) and Fig. 4G and I (duration task). The delta response reflects how much the responses to each level of the relevant magnitude differ from the average response, thus indexing the strength of the serial dependence effect induced by each level of the other dimension. In line with the previous analyses, the different ranges of the task-irrelevant magnitude were assessed independently also in this context.

First, in the numerosity task, the first duration range used in blocks 1, 3, and 5 showed again robust cross-dimension serial dependence effects. As shown in Fig. 4A, the average numerical estimates at each level appear to increase consistently with the increasing duration of the past stimulus, although the effect is not completely linear. Figure 4B better shows the effects at different levels of numerosity in terms of the normalized delta response index. In general, smaller numerosities seemed to be more susceptible to the effect of previous durations compared to larger numerosities, with biases roughly two times stronger at the extreme levels of the previous duration. To assess the significance of the effects, we employed a LME model on the delta response data, with numerosity and duration as fixed effects, and the subjects, as well as numerosity and duration themselves, as random intercepts. The results (adj. *R*^2^ = 0.34) showed a significant effect of both numerosity (*b* = 0.347, *t* = 5.18, *p* < 0.001) and duration (*b* = 0.044, *t* = 6.69, *p* < 0.001), and a significant interaction (b = − 0.001, *t* = − 5.74, *p* < 0.001). We followed up this test with simpler models within each level of numerosity, entering only duration as fixed effect. This series of tests showed a significant effect of past durations at all levels of numerosity (adj. *R*^2^ ≥ 0.16, *b* ≥ 0.012, *t* ≥ 4.20, all *p* < 0.001). To better understand the interaction observed, we compared the effect of the two extreme levels of past duration (150 and 600 ms) across the different numerosities (LME tests with numerosity as fixed effect). In the case of the shorter duration, we observed a reduced effect (i.e., less negative effect) for increasing numerosity (adj. *R*^2^ = 0.39, *b* = 0.338, *t* = 5.57, *p* < 0.001). The longer duration similarly showed a decreasing effect for increasing numerosity (adj. *R*^2^ = 0.16, *b* = 0.172, *t* = − 2.88, *p* = 0.004).

On the other hand, when using the second duration range in blocks 2 and 4, we observed much weaker effects (Fig. 4C, D). In this case, a LME model including both numerosity and duration (adj. *R*^2^ = 0.02) did not show any significant effect (*p* ≥ 0.11) and no interaction (*p* = 0.08). A series of tests within each numerosity level however showed significant effects of past duration at the higher half of the range (16, 24, and 32 dots; adj. *R*^2^ ≥ 0.04, *b* ≥ 0.007, *t* ≥ 2.76,* p* ≤ 0.006) but not at smaller numerosities (8 and 12 dots; *p* ≥ 0.09). Contrary to the first range, here higher numerosities seemed to be more susceptible to biases, although with a much less clear pattern.

In the duration task, we observed noticeable effects of past numerosities (Fig. 4F, G), although weaker compared to the effect of duration on numerosity. When using the first numerosity range, a LME model (adj. *R*^2^ = 0.09) showed only a significant effect of the past numerosity (*b* = 0.479, *t* = 4.76, *p* < 0.001), with no effect of duration (*p* = 0.31) and no interaction (*p* = 0.24). A series of tests within each level of duration further showed significant effects of numerosity at all the duration levels (adj. *R*^2^ ≥ 0.06, *b* ≥ 0.015, *t* ≥ 2.74, *p* ≤ 0.007). Differently from the effect of duration, the effect of past numerosities seemed to be equally effective at different levels of duration. When using the second numerosity range in the duration task (adj. *R*^2^ = 0.003), we instead did not observe any effect of numerosity (*p* = 0.80) or duration (*p* = 0.89), and no interaction (*p* = 0.93). Additionally, we did not observe any significant effect of numerosity across the different levels of duration (*p* ≥ 0.32).

**Fig. 5:**
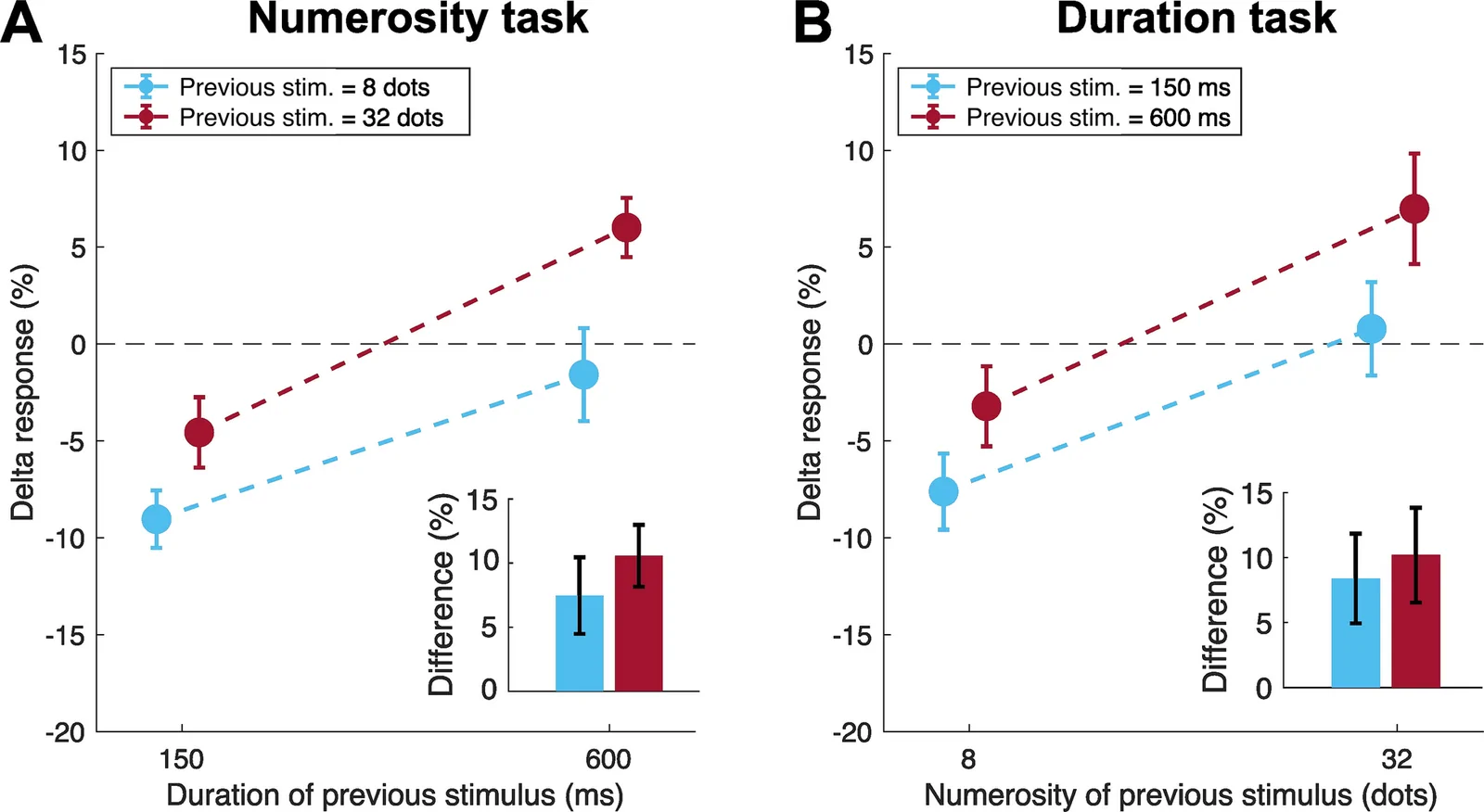
Combination of effects within and across the different dimensions.** A** Numerosity task. Effect of different durations of the past stimulus (limited to the extreme levels) plotted separately according to the numerosity of the previous stimulus (8 or 32 dots; light blue and dark red, respectively). The inset at the bottom of the plot shows the difference between the two pairs of data points. **B** Duration task. Effect of different numerosities of the previous stimulus plotted separately according to the duration of the previous stimulus (150 or 600 ms; light blue and dark red, respectively). The inset shows the difference between the two pairs of data points. Error bars are SEM

Moreover, we focused on different combinations of the magnitudes of the preceding stimulus to assess how the effects within and across dimensions combine with each other. To do so, we first pooled all the trials corresponding to the three intermediate levels of the task-relevant magnitude in the current trial (12, 16, 24 dots; 200, 300, 450 ms). Then, we sorted the trials according to the combination of extreme magnitudes of the preceding stimulus (i.e., 150 ms and 8 dots vs. 600 ms and 8 dots). The results of this analysis are shown in Fig. 5. In both the numerosity (Fig. 5A) and duration (Fig. 5B) task, different types of effect appeared to sum up in an additive fashion. Namely, in the numerosity task, the cross-dimension effect of previous duration is shifted according to the numerosity of the previous stimulus. The same holds true in the duration task. To assess the significance of this modulation, we used two LME test on the delta response measures corresponding to these combinations, including both numerosity and duration as fixed effects, and the subjects, numerosity, and duration, as random intercepts. In the numerosity task, the test (adj. *R*^2^ = 0.23) showed a significant effect of both numerosity (*b* = 0.251, *t* = 3.32, *p* = 0.001) and duration (*b* = -0.020, *t* = − 4.96, *p* < 0.001). Similarly, in the duration task, the test (adj. *R*^2^ = 0.14) showed a significant effect of numerosity (*b* = − 0.387, *t* = − 4.02, *p* < 0.001) and duration (*b* = 0.012, *t* = 2.29, *p* = 0.024). Looking at the difference in delta response caused by duration in the numerosity task (inset in Fig. 5A) and numerosity in the duration task (inset in Fig. 5B), we found similar levels of effect irrespective of the level of numerosity (numerosity task) or duration (duration task) considered. A series of one-sample *t*-tests showed a significant effect of duration in the numerosity task (*t*(27) = 2.50, *p* = 0.019, *d* = 0.47; *t*(27) = 4.37, *p* < 0.001, *d* = 0.83; respectively for 8 and 32 dots) and numerosity in the duration task (*t*(27) = 2.43, *p* = 0.022, *d* = 0.46; t(27) = 2.79, *p* = 0.009, *d* = 0.53; respectively for 150 and 600 ms). Paired *t*-tests showed no significant difference between the effects (*p* = 0.38 and *p* = 0.67, respectively for the numerosity and duration task). These results suggest that effects within and across dimensions add up to each other determining the overall effect measured on the current stimulus. This additional analysis also allowed us to rule out the potential confounding factor of total numerosity, which correlates with average numerosity and duration. If the effect of the previous stimulus were based on total numerosity, then we should have observed a different pattern of effects reflecting the interaction between average numerosity and duration (i.e., as total numerosity increases with both average numerosity and duration).

**Fig. 6:**
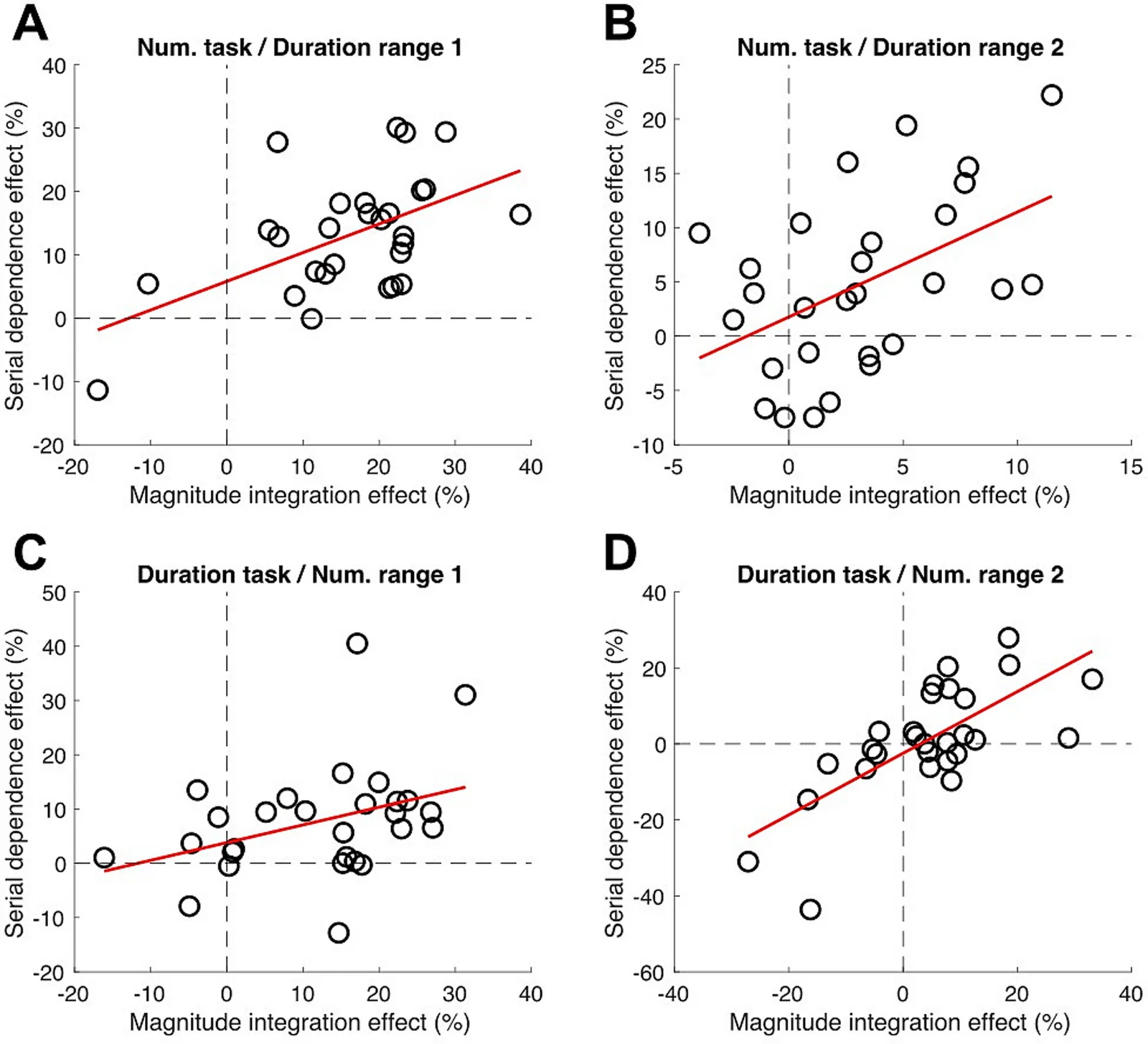
Relationship between serial dependence and magnitude integration effects.** A** Serial dependence effects plotted as a function of the magnitude integration effects, in the numerosity task (duration range 1). **B** Serial dependence effects as a function of the magnitude integration effects, in the numerosity task (duration range 2). **C** Serial dependence effects as a function of the magnitude integration effects, in the duration task (numerosity range 1). **D** Serial dependence effects as a function of the magnitude integration effects, in the duration task (numerosity range 2). The red lines represent a linear fit to the data, added to show the trends. Note that each panel has different ranges on the x and y axes, to better display the trend of the data

Finally, we assessed the relationship between cross-dimension serial dependence effects and magnitude integration effects. To do so, we used the normalized serial dependence indexes (Fig. 3), and computed a similar index of the magnitude integration effect in percentage, based on the normalized difference in the average numerical or duration estimates as a function of the interfering magnitude. We then plotted the magnitude integration effect index as a function of the serial dependence index (Fig. 6), and assessed whether the strength of cross-dimension serial effects could be predicted by the strength of magnitude integration across the sample. To assess the significance of this relationship, we used a LME model within each task, entering the serial dependence effect as dependent variable, the magnitude integration effect and the range (i.e., range 1 vs. range 2) as fixed effects (one continuous, the other categorical), and the subjects, magnitude integration effects, and range as random intercepts. Figure 6A and B show the relationship between the two effects in the numerosity task, separately for the two ranges. Individual linear fits to the data have been added to show the trend of the data. The results of the LME model in the numerosity task (adj. *R*^2^ = 0.94) showed a significant effect of magnitude integration (b = 0.45, *t* = 3.60, *p* < 0.001), no effect of the range (*p* = 0.19), and no interaction (*p* = 0.19). In the duration task (Fig. 6C and D), the LME model (adj. *R*^2^ = 0.99) showed again an effect of magnitude integration (*b* = 0.33, *t* = 2.01, *p* = 0.049) and no effect of range (*b* = *p* = 0.066), but a significant interaction between the two factors (*b* = 0.48, *t* = 2.22, *p* = 0.031). To follow up this interaction, we performed two separate LME tests within each of the two ranges. These tests showed a significant relationship between serial dependence and magnitude integration both in range 1 (adj. *R*^2^ = 0.84, *b* = 0.33, *t* = 2.10, *p* = 0.045) and range 2 (adj. *R*^2^ = 0.93, *b* = 0.81, *t* = 5.34, *p* < 0.001), although the effect seemed weaker in the first range (i.e., *b* = 0.33 compared to 0.81 in the second range), explaining the interaction. Overall, these results show a significant relationship between the two effects, with the magnitude integration effects measured within the same stimulus being able to predict the serial dependence effects across the two dimensions, induced by the preceding stimulus.

In addition to the relationship between different effects, we also assessed the relationship between each of them and the precision in the task (Weber’s fraction; see Additional File 1, Fig. S2-S3 [17, 42–44]). Serial dependence has been indeed often shown to correlate with precision [17, 42–44], raising the question of whether both the effects measured here may show a common relationship with perceptual precision. Our results however show a significant correlation only in the numerosity task (limited to the first duration range), both for serial dependence and magnitude integration. See the Additional File 1, Fig. S2-S3 for more details about these tests.

## Discussion

In this work, we addressed the level of abstraction at which the serial dependence effect originates by leveraging the intrinsic link between different magnitude dimensions. While previous results obtained with static stimuli failed to observe cross-dimension serial dependence between duration and numerosity [30], here we used an optimized setup based on dynamic stimuli and a paradigm more specifically designed to measure effects across magnitudes and their modulation. Our results show for the first time that (1) cross-dimension serial dependence effects do in fact occur between duration and numerosity, and (2) changing the stimulation range across different blocks strongly modulates both serial dependence and magnitude integration effects, in a very similar fashion. Importantly, we also show that (3) the strength of serial dependence can be predicted from the strength of the magnitude integration effect.

First, in terms of general perceptual performance, we observed large overestimation biases of both duration and numerosity. Considering the fast, dynamic nature of the stimuli, some bias in perceived magnitude was indeed expected [42, 43]. However, due to the lack of evidence concerning how strongly a dynamic modulation might affect the perception of our peculiar stimuli, we did not have a precise prediction of how strong such biases might be. Although the observed overestimation seems particularly striking, it is in fact in line with previous results involving dynamic stimuli. Indeed, using dynamic stimuli modulated at relatively high temporal frequencies has been shown to induce large overestimation biases in perceived duration and numerosity. For instance, Kanai and colleagues [45] showed biases of up to 300% in perceived duration with a temporal frequency of 15 Hz (close to our presentation frequency of 20 Hz). In numerosity perception, a temporal frequency of just 8 Hz has been shown to induce an overestimation of up to 35% [46]. The dynamic nature of the stimuli used in our setup thus explains the large, general overestimation biases observed. Besides the effect of the dynamic modulation, such a bias might alternatively (or in addition) reflect (1) a possible influence of total numerosity, which increases with both average numerosity and duration, or (2) a property of the types of judgment performed. In terms of total numerosity, although it is difficult to conclusively rule out a contribution to the observed biases, our results suggest that its influence, if any, is minimal. Indeed, an effect driven by total numerosity predicts a (positive) interaction between average numerosity and duration, which was not observed (see Fig. 5). In terms of the intrinsic properties of the task, in duration reproduction a longer estimate might simply reflect the additional latency needed to perform the action of rising the finger to end the response. In numerosity reproduction, however, such a latency would not be expected, so it is more difficult to explain. In general, since it is difficult to pinpoint the origin of the overestimation bias, our interpretation remains speculative.

Due to the dynamic nature of the stimuli used, other phenomena might possibly be induced by the rapid visual presentation. For example, due to the short timing of each individual array, masking may affect the visibility of the stimuli. However, in a previous study [31] we demonstrated that all arrays along the sequence contribute to the judgment of average numerosity, making the presence of masking unlikely. Apparent motion might also be induced by items in successive displays, depending on their relative position. Due to the random nature of the items’ position in the arrays, apparent motion is not expected to occur systematically and consistently. Even if occurring, it is in any case not expected to affect the magnitude integration or serial dependence bias. Finally, as also mentioned above, although the task involved the estimation of average numerosity, the total amount of dots presented over the sequence represents an additional cue that participants may have used to judge the stimuli. Average and total numerosity are indeed intrinsically correlated, and difficult to disentangle. In a previous study from our group [22], we however demonstrated that total numerosity is a much weaker cue compared to the average. Namely, when keeping the total number of dots constant and varying the duration of the stimuli, we observed that the judgments are strongly biased by the average number of dots, which in that case decreased as the duration of the sequence increased (i.e., longer durations meant that fewer dots were presented on average in each display). Besides this, the response phase of the numerosity task was designed to show numerosities based on the average numerosity range. This makes it difficult for participants to inadvertently judge the total numerosity, as the magnitudes showed when moving the response slider were generally too low to represent the total number of dots. Again, an influence of total numerosity in the observed effect would have also predicted an interaction between duration and average numerosity, which was not observed. Although it is not possible to conclusively disentangle the two dimensions, average numerosity seems nevertheless the dimension that is preferentially computed and more easily judged when using this type of dynamic stimuli.

Compared to our previous study [30], the use of dynamic stimuli seems to greatly facilitate both magnitude integration and serial dependence effects. In line with the interpretation of these effects in previous studies [22, 31], such a facilitation is likely to stem from the use of *average* numerosity over time, making the processing time-course of numerosity much more similar to duration. Indeed, one possible reason limiting the integration of the different magnitudes of static stimuli is the much different processing time-course of temporal and non-temporal dimensions (but see [25, 47] for different explanations). A dimension such as numerosity indeed is usually processed starting from its onset when it is presented spatially (i.e., as an array of items), in a relatively short time spanning around 250 ms after stimulus onset [48–52]. Duration instead needs to fully unfold throughout the interval before the brain could generate a duration representation. Considering this, numerosity can thus interfere with early duration accumulation, while duration can interfere only at much later stages, where numerosity is likely represented in a more stable fashion. Such a difference in the processing dynamics of different dimensions and their different ability to affect an early processing stage can explain the asymmetry often observed in magnitude integration [22, 25]. Namely, duration is typically the dimension showing larger magnitude integration biases, while it usually exerts only a little influence of non-temporal dimensions such as numerosity. Fitting with this interpretation, it has also been shown that a symmetric effect between numerosity and duration can be observed using a very short duration range (~ 50–100 ms; [21]), which likely makes the difference in processing dynamics less pronounced. When using dynamic stimuli, the computation of average numerosity requires an accumulation of information over time [31], and computations akin to the extraction of the summary statistics of a sequence [53, 54], so that a complete representation can only be formed after the offset of the sequence. This is thus more similar to duration, and would facilitate magnitude integration and symmetric effects across the two dimensions. Recent results from our group indeed show that the processing of average numerosity unfolds over time with a peculiar temporal profile, in which earlier information is weighed to a greater degree compared to the later, more recent information (“primacy” effect)—something similar to the computation of summary statistics of successive spatial positions [55].

Since the numerosity estimation task involved a response slider that was kept constant throughout the session (left-to-right magnitude mapping), could the magnitude integration effect be influenced by the compatibility of the response code with the magnitude of the task-relevant and irrelevant dimension? Effects like the spatial-numerical (or spatial–temporal) association of response codes (SNARC or STEARC effect) might have indeed occurred in our setup [35, 36, 40]. These effects are usually measured in terms of response times, with faster responses when the magnitude is consistent with the response code. However, in the numerosity task, the magnitude integration effect induced by duration occurred similarly throughout the range, and even at numerical magnitudes that involved an inconsistent response mapping. For example, a low numerosity is overestimated when paired with a long duration, even if the response side on the slider (the left part) is inconsistent with the long duration (which would be mapped on the right). Our results (see Additional File 1, Fig. S1) also show no difference in response times as a function of numerosity or duration. Finally, very similar effects were observed in the duration task, whereby only one central button was used by participants to judge the stimuli. In this setup, the response code was thus neutral when it comes to its compatibility with magnitude, and thus no compatibility effect is expected. These results suggest that the potential association between magnitude and space introduced by the response slider did not play a significant role in driving the serial dependence and magnitude integration effect. However, due to the nature of the tasks used (especially the numerosity task), response-compatibility effects might still interact with the magnitude judgements in ways that we could not capture with our analyses focused on specific effects. Addressing the phenomenon of response-compatibility in our specific tasks remains an open question for future studies.

In terms of serial dependence, we observed robust biases both within and across each dimension. The effect within each dimension seems to be more strongly based on the response in the previous trial, rather than the previous stimulus itself. This might suggest that past “decisions” play a more pronounced role in inducing serial dependence, in line with previous accounts of the effect [13, 41, 56]. However, considering both the general estimation biases observed in our results (i.e., the systematic overestimation of numerosity and duration) and the magnitude integration bias itself, the response in the previous trial also more closely reflects how the stimulus was *perceived*. There is indeed evidence that serial dependence works according to the perceived properties of past stimuli (i.e., for instance when affected by visual illusions; [57, 58]). Thus, the effect of previous responses might reflect the perception of the previous stimulus rather than the decision made based on it, also considering that a response is not strictly necessary to observe an effect (e.g., [59]; see also discussion below). However, note that this reasoning is valid only assuming that the observed biases in perceived magnitude are in fact perceptual biases. If so, then the response would more accurately index how the stimulus was perceived. Otherwise, if the biases are driven by post-perceptual processes, the response would more closely reflect decisional processes rather than perception. Considering the previous evidence mentioned above, if the overestimation bias is driven by the dynamic nature of the stimuli this would support a closer link between the response and perception. However, based on behavioral results alone it is difficult to disentangle the different possible contributions to this bias, and hence this point remains speculative.

We also observed strong cross-dimension biases, working according to the previous stimulus magnitude (since the irrelevant magnitude was never explicitly judged). Considering the complexity of the task and the systematic, concurrent modulation of the different dimensions, it seems that the brain relies on an integrated representation of perceptual history, rather than on a single stimulus dimension. The cross-dimension effects thus also suggest that serial dependence originates from a processing level sufficiently high to allow the interaction and integration of different dimensions. Potentially, a processing level whereby perceptual information has been extracted from its original input properties and represented in an abstract fashion. This is consistent with previous observations showing some degree of abstraction of the serial dependence effect. For instance, Fischer et al. [16] demonstrated that the contextual properties of a stimulus, such as the color of a grating stimulus, have little effect on the strength of serial dependence. In numerosity perception, it has also been shown that serial dependence can work across completely different stimuli, like a sequence of events and an array of dots, or even from a symbolic numeral to an array of dots [17, 18]. These results thus converge together in showing that serial dependence originates from relatively high-level computations [56–58, 60]. Importantly, however, our results show for the first time that such a high-level, abstract nature not only makes the effect invariant to low-level features, but also gives rise to effects across different dimensions. See below for more discussion about the potential nature of serial dependence effects in this context. Our additional results concerning the relationship between the different effects and the precision in the task (Weber’s fraction, see Additional File 1, Fig. S2-S3) however show a partial dissociation between effects occurring in different tasks. Indeed, only the effects in the numerosity task (both magnitude integration and serial dependence) show a correlation with precision (the higher the effect, the better the precision). Thus, although potentially reflecting information from an abstract magnitude processing stage, both effects have a task-specific component.

How do the different magnitudes involved interact with each other? Our analysis shows that in the numerosity task the effectiveness of past durations depends on the numerosity in the current trial. Smaller numerosities (8–16 dots) appear to be more prone to cross-dimension serial dependence effects, while larger numerosities are less susceptible to the effect. This appears to be in contrast with previous results, showing weaker effects at smaller numerical magnitudes [2, 15]. However, in the second, larger duration range used in the second and fourth block of the experiment, we did not observe such a pattern. In the duration task, conversely, we did not observe any effect of the duration in the current trial. The data in this condition however also appears noisier, so that the additional variability may potentially make finding a pattern more difficult. In terms of the combination of different magnitudes of the previous stimulus, we observed additive effects. Namely, the effect of numerosity and duration seem to add up to each other, in line for instance with the combination of different effects observed in [30]. This is interesting, as it also suggests that within-dimension and cross-dimension effects occur simultaneously, determining together the overall bias observed on the current stimulus. In other words, both the task-relevant and task-irrelevant dimensions of the previous stimulus are similarly effective in inducing serial dependence. It is important to note however that the analysis of different individual combinations of magnitudes is limited by the relatively low number of trials used in the study (see also below for more discussion about this), so that interpreting these results needs caution. Indeed, this additional analysis required selecting specific combinations of stimuli that were presented only in a limited number of trials, while our more general analyses (e.g., Fig. 3) involved the pooling of larger numbers of trials.

Moreover, our results show that modulating the magnitude range presented across different blocks concurrently affects the strength of magnitude integration and serial dependence. Indeed, while the effects are robust when using the first range in blocks 1, 3, and 5 of the experiment, both magnitude integration and serial dependence show a much different pattern in blocks 2 and 4. Such a modulation was expected based on our hypothesis of a calibration of magnitude perception, although the observed effect is much stronger than we might have predicted. Indeed, both effects were strongly reduced, or even completely abolished. Why does changing the range reduce the effects across magnitudes? In our original design, we hypothesized that magnitude integration would require a calibration to the specific statistics of the stimuli, occurring with exposure to the stimuli at the beginning of the experiment and potentially persisting even when switching range—a hypothesis that we are currently testing with additional dedicated experiments. This idea, at its core, is driven by the relative nature of the magnitude integration effect, showing biases as a function of the specific stimuli presented in an experiment rather than the absolute magnitude values. Namely, similar durations can induce either under- or over-estimation depending on whether they represent the lower or higher part of the range used. For example, 150 ms causes underestimation in the present study, while ~ 100 ms caused overestimation in [21]. Similarly, 31 dots induced an underestimation in [21], while an average of 32 dots induce an overestimation in the present study and in [23]. Other studies used a variety of different ranges, so that no individual magnitude level is linked to a specific underestimation or overestimation effect in absolute terms [24, 61, 62]. Interpreting our results in light of this hypothesis, our findings suggest that serial dependence, similarly to magnitude integration, may undergo a calibration to the stimulation statistics, and that it may not be easily recalibrated when such statistics suddenly change.

Another possibility is that the different ranges used are not optimal to induce magnitude integration and serial dependence effects. Indeed, when changing range, we switched to both higher numerosities (24–96 dots instead of 8–32) and longer durations (450–1200 ms instead of 150–600 ms). Although it is possible that such ranges may be less optimal, this would be in contrast with previous studies using similarly large magnitude ranges to test integration effects. Indeed, similar long duration ranges have been used successfully in previous studies (i.e., 600–1000 ms in [24]; 400–1600 ms in [61]; 800–1200 ms in [62]). In terms of numerosity, Javadi & Aichelburg [21] used a range that sits in the middle of the ones used here (28–40 items), and successfully observed magnitude integration effects. New data from our group also show significant serial dependence effects at higher magnitudes, up to 64 dots and 800 ms (Togoli et al., under review). Besides the evidence from previous studies suggesting that higher magnitudes should be effective in inducing serial dependence and magnitude integration, the present results more directly (albeit partially) support a role of the presentation order and calibration. Namely, since the two ranges partially overlapped, we should expect the shared magnitude levels (24–32 dots, 450–600 ms) to also be effective in the second range. If the higher magnitudes fail to induce a bias, then a reduction in the strength of the effect is expected mostly at those levels. However, our results show an almost complete lack of effect in the second range, at all magnitude levels. Comparing for instance Fig. 2A and C with Fig. 2B and D, it is clear that the same magnitude levels induce different effects: very strong when included in the first range, virtually zero in the second range. This observation supports the idea that it is the calibration to the overall range that limits the effects, and not the chosen magnitude levels being less effective. Nevertheless, although a calibration effect seems more plausible considering previous and current evidence, the fixed order of the different magnitude ranges represents a limitation that should be taken into account. More evidence is thus required to draw a firmer conclusion on this point, and to better understand the role of calibration and the effect of the presentation order. Irrespective of the reason leading to the reduced effect, what is interesting is that both magnitude integration and serial dependence are similarly reduced, suggesting a link between the two effects.

Stronger evidence for a link between the two phenomena is however provided by the significant relationship between their strength. Indeed, our results show that the strength of serial dependence across the group can be predicted by the strength of the magnitude integration effect. Namely, participants showing stronger magnitude integration are likely to also show stronger serial dependence, and vice versa—irrespective of the range used. How are these two seemingly different phenomena linked? One possibility is that the link may reflect the same information (i.e., abstract magnitude representations) being used for different purposes, i.e., first used within a stimulus, and then carried over to the next inducing serial dependence. However, using the same information per se does not fully explain such a link, and it is thus possible that the two phenomena may also share similar mechanisms. For instance, it is possible that they may be both implemented through similar integration mechanisms, working according to the same computational structure. That is, a more general integration mechanism gathering all available information, both present and past, to support the representation of a magnitude dimension. At the neural level, a possibility is that both phenomena could arise from the interaction of partially overlapping neural populations encoding different magnitudes, as it has been observed in brain regions like the parietal cortex [63–65]. These possibilities however remain speculative, and further studies at the neural level are needed to better understand the mechanisms involved. Addressing the possibility of a shared mechanism mediating magnitude integration and serial dependence represents another interesting direction for future studies.

In terms of serial dependence, a possibility is that the effects observed here might reflect a perceptual “stability” process. That is, an integration of past and present magnitude information that allows to smooth out noise from neural signals and perceptual representations. Serial dependence indeed has been hypothesized to play a role in aiding perceptual stability and continuity [5, 66–68]. This account is however debated, and an alternative framework posits that serial dependence in fact involves decisional rather than perceptual processes [13, 56]. Even if often pitted against each other, these two accounts need not to be mutually exclusive, as serial dependence might arise from distinct independent mechanisms implemented at different processing levels, engaged depending on the stimuli and task used. In the case of magnitude perception, although an “abstract” effect across different dimensions might suggest a post-perceptual mechanism, previous results show that the link between magnitudes entails perceptual processes. Indeed, we have recently shown that information from different magnitude dimensions is integrated at the perceptual level, relatively early in the brain processing stream, and even in the absence of a task [69]. This supports the idea that, at least in this case, the mechanism involved might be implemented at mid- or high-level visual (perceptual) stages, and potentially related to perceptual stability. In this context, magnitude integration within the dimensions of the same stimulus might serve a similar purpose. That is, achieving a more robust representation by leveraging the potential correlation between magnitudes. However, this interpretation remains purely speculative. Our behavioral data do not allow to draw a firm conclusion on the nature of the mechanisms involved, which is thus another question that remains open.

Finally, a limitation of the present study is the use of a paradigm optimized for the testing of magnitude integration and its calibration rather than serial dependence. Although our design provided the opportunity to assess the modulation of the effects across different blocks, the stimulation structure resulted in having fewer trials in the serial dependence analysis compared to previous studies. While the number of trials available in our main analysis (Fig. 3B and D) is similar to the number of repetitions used in previous studies in numerosity and magnitude perception [8, 17, 32, 58, 60], it is much lower compared to studies in orientation perception, typically involving thousands of trials [5, 13, 41, 70]. Although this certainly represents a limitation that should be considered, our results nevertheless show robust effect sizes for the cross-dimension effects, with Cohen’s d values spanning from ~ 0.7 (duration task) to ~ 1.3 (numerosity task). This suggests that when using a paradigm optimized to facilitate the relationship between different magnitudes, the cross-dimension effects are robust despite the relatively low number of trials. As mentioned earlier in the discussion, the low number of trials still limits the analysis of the different individual combinations of magnitudes in the past and present trials. A larger dataset may thus be needed to more accurately address the interaction of different magnitudes and different effects (i.e., serial dependence and magnitude integration).

## Conclusions

To conclude, our findings demonstrate for the first time that serial dependence can occur across two different perceptual dimensions. This in turn supports the idea that serial dependence originates from mid-level or high-level computations in which the information is abstracted from the low-level properties of the stimuli and its origin. While previous studies supported this idea by showing an invariance of the effect to changes in low-level properties, here we demonstrate that the abstract nature of the effect also entails biases crossing from one perceptual dimension to another, at least in magnitude perception. Finally, we demonstrate that serial dependence across successive stimuli can be predicted based on magnitude integration effects occurring within each stimulus, suggesting a link between the two phenomena and, potentially, the existence of shared mechanisms or computations. Overall, our findings contribute to better establishing the properties of serial dependence and the nature of the brain computations giving rise to this effect.

## Methods

### Participants

The study included a total of 30 participants (sex: 21 females and 9 males), with age ranging from 18 to 38 (mean age ± SD = 25.1 ± 5.7). All participants had normal or corrected-to-normal visual acuity, and were naïve to the purpose of the experiment. Before participating in the study, each participant read and signed an informed consent form. The reward for participation was 25 Euros. Two participants were excluded from data analysis due to insufficient performance (i.e., Weber’s fraction > 1), leaving a total of 28 participants included in the final sample. The study was approved by the ethics committee of Université Catholique de Louvain (protocol #2023–48), and was designed to be in line with the declaration of Helsinki. The sample size tested in this study was decided a priori based on a power analysis. The power analysis was performed according to the expected effect size of the magnitude integration effect measured in previous studies from our group using similar stimuli [22]. Namely, we computed the expected effect size (Cohen’s d) by averaging the minimum effect size of the bias of duration on numerosity and the minimum effect size of numerosity on duration (i.e., effect of the smaller magnitude difference levels in Exp. 1b of [22]). The resulting effect size was *d* = 0.76. By using a two-tailed distribution and a desired power of 95%, the expected estimated sample size was 25, which we rounded up to 30 to be more conservative and account for the possibility of data exclusion.

### Stimuli

The visual stimuli were generated using the Psychophysics Toolbox [71, 72] in Matlab (r2021b, The Mathworks, Inc.). In the experiment, the stimuli were displayed on a 1920 × 1080 LCD monitor running at 120 Hz, encompassing a visual angle of approximately 48 × 30 degrees of visual angle from a distance of 57 cm. The experiment was performed in a quiet and dimly lit room, with the only source of light being the monitor screen of the experimental setup, to avoid distractions. The design and structure of the stimuli was based on previous studies from our group [22, 31]. Each stimulus was composed by a sequence of multiple, briefly-presented (50-ms each; 20 Hz frequency) arrays of dots, modulated in average numerosity (i.e., the average number of dots computed across all the arrays included in the stimulus sequence) and in duration (the total duration of the sequence, which corresponds to the number of individual 50-ms arrays included). The modulation of average numerosity was achieved by having the number of dots in each array varying around the mean numerosity of the sequence selected in each trial (± 50% around the base value of average numerosity). The numerosity of each array in a sequence was computed ad-hoc before the stimulus presentation in order for the sequence to result in a specific average numerosity, while we kept the variance of each sequence similar irrespective of the number of the individual arrays. This stimulus design and the fast presentation frequency (20 Hz) were chosen specifically so that each sequence would be more likely perceived as a fast dynamic stimulus rather than a series of static dot arrays, while avoiding too much perceptual “overlap” between consecutive arrays [31]. The location of each dot in a single array was computed to avoid overlapping with other dots, and considering a minimum distance between any pair of dots of 2.5 times the radius of an individual dot (dot radius = 6 pixels, or 0.14°; minimum inter-dot distance = 15 pixels, or 0.35°). The circular area in which the dots were drawn had a radius of 250 pixels (approximately 5°). Each array in the sequences had the same area and the same dot size. The dots in each array were 50% black and 50% white, presented on a grey background (luminance values: black = 0.24 cd/m^2^, grey = 46.79 cd/m^2^, white = 98.17 cd/m^2^).

The choice of using a task based on the average numerosity rather than total numerosity of the sequences is mainly driven by previous results from our group comparing these tasks. Indeed, we previously observed that the judgment of total numerosity is strongly affected by the average number of dots shown in the sequence, but not vice versa [22]. Additionally, the judgment of average numerosity showed higher precision (i.e., lower Weber’s fraction) compared to total numerosity ([22]; see Additional File 1, Fig. S2-S3). This suggests that the average numerosity is a feature that the visual system has a more direct access to, and/or is more sensitive to, making the task easier. Even if the judgment of average numerosity seems easier compared to total numerosity, an important property of our stimuli that needs to be taken into account is the intrinsic correlation between the different dimensions. Indeed, total numerosity correlates positively with average numerosity (the larger the number of dots presented on average, the larger the total number of dots presented over the entire sequence), and with duration (the longer the duration, the larger the total amount of dots presented). In terms of numerosity per se, our paradigm could not dissociate different numerical cues. However, the response procedure was devised to display numerosities based on the average numerosity range, making it unlikely for participants to get confused between the two numerical properties and inadvertently judge total numerosity (i.e., the quantities displayed during the response phase were generally too small to represent the total numerosity of the sequences). In terms of effects, total numerosity could contribute to the effect of average numerosity and duration, both considering serial dependence and magnitude integration. However, since total numerosity increases with both average numerosity and duration, any effect of this alternative cue would thus be based on their combination. We addressed this possibility with specific analyses assessing the combined effect of different magnitudes (see below Data analysis). Indeed, any combined effect that could be explained by total numerosity should show an interaction between average numerosity and duration. For instance, a serial dependence effect purely based on total numerosity should show a very strong amplification when a numerosity is coupled with a long duration, beyond what could be expected from the effects of average numerosity and duration per se.

The experiment included two task conditions performed in separate sessions by the same participants, in which we used similar stimuli but with different levels of either duration or numerosity. Indeed, while the range of the task relevant dimension in each task was kept constant throughout the session, the range of the task-irrelevant, interfering dimension was alternated in different blocks (each session included 5 blocks of trials; see below Procedure). In the numerosity task condition, the average numerosity of the stimulus sequence could be 8, 12, 16, 24, or 32 dots. In block 1, 3, and 5, the duration of the stimuli could be 150, 200, 300, 450, or 600 ms (corresponding to 3, 4, 6, 9, or 12 individual 50-ms arrays composing the sequence, respectively; duration range 1). In block 2 and 4 of the session, instead, the duration could be 450, 600, 750, 900, or 1200 ms (corresponding to 9, 12, 15, 18, or 24 individual arrays; duration range 2). In the duration task, the duration of the stimuli could be 150, 200, 300, 450, or 600 ms (3, 4, 6, 9, or 12 individual arrays). The average numerosity in blocks 1, 3, and 5 could be 8, 12, 16, 24, or 32 dots (numerosity range 1). In blocks 2 and 4, the average numerosity could be 24, 32, 48, 68, or 96 dots (numerosity range 2). This manipulation was aimed at introducing a modulation of the effect, and at assessing the flexibility of both the magnitude integration effect (within each stimulus) and the serial dependence effect (across successive stimuli) in face of changes in the stimulation range, as well as the relationship between the two effects. Specifically, we hypothesized that once calibrated to a specific range of magnitude, the integration system may not be flexible enough to recalibrate to a different range, reducing the effectiveness of the interfering dimension. We thus expected at least a reduced effect when switching range, due to the overlap between the lower and upper portion of the two ranges and the need to recalibrate the effect to the new range. This potential modulation could thus be leveraged to further assess the relationship between magnitude integration and serial dependence effects, and their mechanisms. All the magnitude ranges were chosen to have a roughly equal span in a logarithmic (log2) space (i.e., to span ± 1 logarithmic unit around the central value). Note that to increase the efficiency of the experiment and facilitate data analysis, we chose to present the two magnitude ranges with a fixed order across participants. In doing so, when computing the effects and averaging the data, we ensured that the same magnitude levels are presented in corresponding blocks. Counterbalancing the order of the different ranges would have required to test two independent samples, to avoid averaging together effects induced by different magnitude levels. Results from previous studies however suggest that the two ranges selected should be similarly effective in inducing effects, so that, in principle, they are not expected to result in widely different biases (e.g., [21–24, 62, 63]).

### Procedure

The experiment involved two task conditions, performed by the same participants in two separate sessions (within the same day). The structure of the two task conditions was primarily designed to measure magnitude integration effects across duration and numerosity, their flexibility in face of changes in the stimulation range, and their evolution and calibration across different trials. Although not optimized for serial dependence, the paradigm was however suitable for serial dependence effects to occur, and the modulation of the magnitude integration effect via different ranges of the interfering dimension provided the opportunity to better assess the relationship between the two phenomena. In both task conditions, the session included 5 blocks of 100 trials, in which we alternated different ranges of the task-irrelevant dimension (i.e., duration in the numerosity task, numerosity in the duration task). Each block was further divided into 20 mini-blocks of 5 trials each. In each mini-block, we presented the entire range of the task-relevant dimension (5 levels, one per trial, with their order pseudo-randomly shuffled), while keeping the task-irrelevant dimension constant. Namely, each of the five trials in a mini-block had a different level of the task-relevant dimension, and the same level of the irrelevant one. The full range of the task-irrelevant dimension was instead presented throughout each series of 5 consecutive mini-blocks (i.e., 25 trials). For example, in the numerosity task, a given mini-block had all the five levels of numerosity, and one level of duration. In the next mini-block there will be again all the five levels of numerosity, and a different level of duration. And so on, in order to also have all the levels of duration tested in a series of five mini-blocks. Within each block, each level of the task-relevant dimension was thus repeated 20 times, while each level of the task-irrelevant dimension was repeated 4 times.

A schematic depiction of the experimental procedure is shown in Fig. 1. Each trial started with the participant keeping their gaze on a central fixation cross. The stimulus sequence, comprising a variable number of dot-arrays modulated in numerosity, was then presented centrally on the screen for a variable amount of time depending on the number of arrays selected (150–600 ms/450–1200 ms in the numerosity task; 150–600 ms in the duration task). The offset of the sequence was followed by a 600-ms blank interval, and then by the response phase. In the numerosity task, a slider appeared at the bottom of the screen, which participants were instructed to use (by moving the computer mouse) to produce an array matching their perception of the average numerosity of the sequence. Moving the slider from left to right linearly increased the numerosity of a static array of dots appearing at the center of the screen, with the minimum and maximum numerosity selectable equal to 1 and 40, respectively. In each trial of the numerosity task, the starting position of the cursor on the slider was determined randomly to avoid response biases. After clicking the mouse button to confirm the response, the next trial started automatically after an inter-trial interval of 1050 ms. In the duration task, participants were instructed to reproduce the duration of the interval by pressing and holding the space bar on the computer keyboard. In the response phase, the fixation cross turned blue, indicating the time to initiate a response. After releasing the button at the end of the reproduced interval, the next trial started automatically after 1050 ms.

### Data analysis

First, we assessed the numerosity and duration reproduction performance and the magnitude integration effect. To do so, we averaged the numerical or duration reproduction responses corresponding to each level of the relevant dimension in each task. This was done by additionally separating the responses in which the task-irrelevant dimension was higher or lower than the central value of the specific range. Different ranges of the task-irrelevant dimensions were kept separated in order to assess a potential modulation of the magnitude integration effect occurring when switching magnitude range. The significance of the magnitude integration effect was assessed by comparing the average numerical or duration estimates as a function of the task-irrelevant magnitude. Based on the typical effect of magnitude integration, numerosity should be overestimated when the stimulus has a long duration, and underestimated when it has a short duration (i.e., longer/shorter than the central value of the range), and the same for the effect of numerosity on duration (i.e., more/less numerous). The average responses across the range, sorted according to the irrelevant dimension, were then assessed with a linear mixed-effect (LME) model. In the model, we entered the average responses of each participant as the dependent variable, and the average numerosity and sequence duration as fixed effects. Random effects were additionally included representing the subjects, as well as duration and numerosity (i.e., random intercepts):

$$\begin{aligned}&\mathrm{Average}\;\mathrm{response}\;\sim\;\mathrm{Numerosity}\;\times\mathrm{Duration}\;\\&+\;(1\left|\mathrm{Subj}.\right.)\;+\;(1\left|\mathrm{Numerosity}\right.)\;+(1\left|\mathrm{Duration}\right.)\end{aligned}$$

In the numerosity task, magnitude integration would thus be indexed by a significant effect of duration in the LME model, and vice versa in the duration task. The overall performance in the tasks was assessed by computing the Weber’s fraction (WF), that is, the variability (standard deviation) of responses divided by the average response, at each numerosity and duration level (respectively for the numerosity and duration task). A cut-off for participant exclusion was set at WF > 1, to exclude participants showing insufficiently precise performance. WFs across the two ranges and task conditions were compared using a LME model. The model included the WF in the numerosity task as dependent variable, the WFs in the duration task and the range as fixed effects, and the subjects as the random effect.

The serial dependence effect was assessed both within each dimension (i.e., effect of previous numerosity on current numerosity judgement, and the same for duration), and across the two dimensions (i.e., effect of duration on numerosity, and vice versa). The within-dimension effects were first computed by taking all the trials in which the central level of the task-relevant magnitude was presented (16 dots or 300 ms, respectively for the numerosity and duration task), and then sorting them according to whether (1) the preceding stimulus had either a lower or a higher level of the relevant magnitude (i.e., 300 ms preceded by 150 ms vs. 300 ms preceded by 600), or (2) the previous reproduction response was either lower or higher than the average of all responses. We then averaged the responses to the stimuli sorted according to the preceding magnitude or responses. Doing so, we thus obtained an index of the serial dependence effect as a function of both the previous stimulus and the previous response (see for instance Pascucci et al. [13]). In the latter case, we used the average response as criterion to sort the trials since we did not assume that the accuracy of responses would reflect the veridical magnitude of the stimuli (i.e., especially in the duration task we expected large overestimations, since sub-second durations are very difficult to reproduce accurately). The cross-dimension serial dependence effects were computed in a similar way, but only limited to the magnitude of the preceding stimuli (i.e., as no response was provided to the task-irrelevant dimension in each condition). Namely, we took all the trials in which the central level of the task-relevant magnitude was presented, and divided them according to whether the task-irrelevant magnitude in the preceding trial was lower or higher than its central value. For example, in the numerosity task, we took all the trials in which an average of 16 dots was presented, and sorted them according to whether the duration of the previous trial was shorter (e.g., 150–200 ms) or longer (e.g., 450–600 ms) than the middle level. The opposite was done for duration and numerosity in the duration task. This analysis was performed separately for the different ranges of the task-irrelevant dimension (i.e., duration/numerosity range 1 in blocks 1, 3, 5, and range 2 in blocks 2 and 4). We then averaged the responses to the stimuli sorted in this way. To better assess the strength of both within-dimension and cross-dimension serial dependence effects and test their significance, we computed a normalized index in percentage similar to previous studies [8, 30], according to the following formula:

$$\begin{aligned}&\mathrm{Serial}\;\mathrm{dependence}\;\mathrm{effect}\;\mathrm{index}\;\\&=\;(({{\mathrm{AvgResp}}_{\mathrm H}\;-\;{\mathrm{AvgResp}}_{\mathrm L})/({\mathrm{AvgResp}}_{\mathrm L})\times100;}\end{aligned}$$

where AvgResp_H_ reflects the average response when the stimulus is preceded by a higher magnitude, and AvgResp_L_ when it is preceded by a lower magnitude. The significance of the effects computed in this way was assessed using one-sample *t*-tests against zero. The analysis of within-dimension effects was performed using on average (± SD) 99.6 ± 0.48 trials. Due to the structure of the experiment and the combination of the different magnitudes in each mini-block, the cross-dimension serial dependence analysis was instead performed using an average (± SD) of 47.8 ± 1.64 trials for range 1, and 31.4 ± 1.89 trials for range 2. The relationship between effects in the two tasks was assessed with a series of LME models. For the within-dimension effects, we performed two LME tests including either the effect in terms of previous stimuli or previous responses. In these models, we entered the serial dependence effect in the numerosity task as the dependent variable, the effect in the duration task as fixed effect, and the subjects as the random effect. For the cross-dimension effect, we entered the serial dependence effect in the numerosity task as the dependent variable, the effect in the duration task and the range as fixed effects, and the subjects as the random effect.

Additionally, we performed a more thorough analysis of cross-dimension serial dependence effects by considering all the possible combinations of task-relevant (the “current” stimulus) and task-irrelevant (the “past” stimulus) dimensions. Namely, we sorted the trials according to each unique combination of the levels of the task-relevant magnitude as a function of each level of the task-irrelevant magnitude in the preceding trial. Responses were then averaged separately for each unique combination. To better visualize and compare the serial dependence effects, we considered separately the combinations of each level of the task-relevant magnitude with all the levels of the task-irrelevant magnitude. Based on this we computed a normalized measure of “delta response” as the difference between the judgements and the mean response to all stimuli with a specific level of the task-relevant magnitude, according to the following formula:

$$\mathrm{Delta}\;\mathrm{response}\;=(({\mathrm{AvgResp}}_{\mathrm J}\;-\;{\mathrm{AvgResp}}_{\mathrm M})/({\mathrm{AvgResp}}_{\mathrm M})\times100;$$

Where AvgResp_J_ reflects the mean response of each combination of one specific level of the task-relevant magnitude, and AvgResp_M_ reflects the mean response of all the combinations of the specific level of the task-relevant dimension and all the levels of the preceding task-irrelevant dimension. Again, these indexes were computed separately for the different ranges of the task-irrelevant magnitude in blocks 1, 3, and 5 and blocks 2 and 4. To assess the significance of the effects, we used a LME model including the delta response as dependent variable, numerosity and duration as fixed effects, and the subjects as well as numerosity and duration themselves as random intercepts. Interactions between numerosity and duration were followed up with simpler models within each dimension.

We then focused on specific combinations of the magnitudes of the preceding stimuli to assess how effects within and across each magnitude combine with each other. To do so, we pooled all the trials in which the three intermediate levels of the task-relevant magnitude were presented in the current trial (12, 16, 24 dots; 200, 300, 450 ms; respectively for the numerosity and duration task), and sorted them according to the combination of magnitudes in the preceding trial. Namely, in both tasks we took the combinations of the extreme magnitudes in the preceding stimulus: 8 dots / 150 ms, 8 dots / 600 ms, 32 dots / 150 ms, and 32 dots / 600 ms. The “delta response” measure was calculated as explained above for these combinations of stimuli. We then used a regression model to assess whether the effect of the irrelevant magnitude differs according to the specific level of the relevant magnitude in the preceding trial. For instance, in the numerosity task, we tested the difference between the effect of 150 and 600 ms as a function of whether they were coupled with 8 or 32 dots. The regression model included the delta response as dependent variable, numerosity and duration of the preceding stimulus as fixed effects, and random intercepts representing the subjects and the numerosity and duration. Further tests were performed on the difference between the delta response corresponding to different combinations (one-sample and paired *t*-tests). This streamlined analysis also served to disentangle the correlation between total numerosity and duration. That is, if the effect is purely based on total numerosity, we should observe an interaction between the two dimensions.

Finally, we assessed the relationship between the magnitude integration effect and the cross-magnitude serial dependence effect. To do so, we employed a LME model aimed at assessing whether the strength of serial dependence effects across the group could be predicted by the magnitude integration effect. The test was performed separately in each of the two task conditions, but including both ranges of the task-irrelevant dimension together as additional (categorical) factors, according to the following model:

$$\begin{aligned}&\mathrm{Serial}\;\mathrm{dep}.\;\sim\;\mathrm{Mag}.\;\mathrm{Integration}\;\times\;\mathrm{Range}\;\\&+\;(1\left|\mathrm{Subj}.\right.)\;+\;(1\left|\mathrm{Mag}.\;\mathrm{integration}\right.)\;+\;(1\left|\mathrm{Range}\right.)\end{aligned}$$

Interactions between the magnitude integration effect and the range were followed up with additional simpler tests within each range.

All analyses and statistical tests were performed in MATLAB (The Mathworks, Inc.; version r2023a).

## Supplementary Information

Additional file 1: Additional analyses concerning the spatial-numerical association effect and relationship between the effects and the precision in the task.

## Data Availability

All data generated and analysed during this study and the experimental code used to perform the experiment are freely available on Open Science Framework at this link: https://osf.io/yfb7r (DOI: https://doi.org/10.17605/OSF.IO/YFB7R).

## Abbreviations

LME: Linear mixed-effect model
SNARC: Spatial-numerical association of response codes
STEARC: Spatial-temporal association of response codes
WF: Weber’s fraction

## References

[1] Kohn A. Visual adaptation: physiology, mechanisms, and functional benefits. J Neurophysiol. 2007;97(5):3155–64. 10.1152/jn.00086.2007. 17344377

[2] Cicchini GM, Anobile G, Burr DC. Compressive mapping of number to space reflects dynamic encoding mechanisms, not static logarithmic transform. Proc Natl Acad Sci U S A. 2014;111(21):7867–72. 10.1073/pnas.1402785111. PMC4040572 24821771

[3] Manassi M, Murai Y, Whitney D. Serial dependence in visual perception: a meta-analysis and review. J Vis. 2023;23(8):18. 10.1167/jov.23.8.18. PMC10476445 37642639

[4] Pascucci D, Tanrikulu ÖD, Ozkirli A, Houborg C, Ceylan G, Zerr P, et al. Serial dependence in visual perception: a review. J Vis. 2023;23(1):9. 10.1167/jov.23.1.9. PMC9871508 36648418

[5] Fischer J, Whitney D. Serial dependence in visual perception. Nat Neurosci. 2014;17(5):738–43. 10.1038/nn.3689. PMC4012025 24686785

[6] Cicchini GM, Mikellidou K, Burr DC. Serial dependence in perception. Annu Rev Psychol. 2024;75(1):129–54. 10.1146/annurev-psych-021523-104939. 37758238

[7] Barbosa J, Compte A. Build-up of serial dependence in color working memory. Sci Rep. 2020;10(1):10959. 10.1038/s41598-020-67861-2. PMC7331714 32616792

[8] Fornaciai M, Park J. Serial dependence in numerosity perception. J Vis. 2018;18(9):15. 10.1167/18.9.15. PMC6141228 30242385

[9] Alais D, Leung J, Van der Burg E. Linear summation of repulsive and attractive serial dependencies: orientation and motion dependencies sum in motion perception. J Neurosci. 2017;37(16):4381–90. 10.1523/JNEUROSCI.4601-15.2017. PMC6596560 28330878

[10] Liberman A, Fischer J, Whitney D. Serial dependence in the perception of faces. Curr Biol. 2014;24(21):2569–74. 10.1016/j.cub.2014.09.025. PMC4254333 25283781

[11] Van der Burg E, Toet A, Brouwer AM, Van Erp JBF. Serial dependence of emotion within and between stimulus sensory modalities. Multisens Res. 2021;35(2):151–72. 10.1163/22134808-bja10064. 34592713

[12] Motala A, Zhang H, Alais D. Auditory rate perception displays a positive serial dependence. Iperception. 2020;11(6):204166952098231. 10.1177/2041669520982311. PMC7758668 33425315

[13] Pascucci D, Mancuso G, Santandrea E, Della Libera C, Plomp G, Chelazzi L. Laws of concatenated perception: vision goes for novelty, decisions for perseverance. PLoS Biol. 2019;17(3):e3000144. 10.1371/journal.pbio.3000144. PMC6400421 30835720

[14] Collins T. The perceptual continuity field is retinotopic. Sci Rep. 2019;9(1):18841. 10.1038/s41598-019-55134-6. PMC6906435 31827138

[15] Fornaciai M, Park J. Attractive serial dependence between memorized stimuli. Cognition. 2020;200:104250. 10.1016/j.cognition.2020.104250. 32155467

[16] Fischer C, Czoschke S, Peters B, Rahm B, Kaiser J, Bledowski C. Context information supports serial dependence of multiple visual objects across memory episodes. Nat Commun. 2020;11(1):1932. 10.1038/s41467-020-15874-w. PMC7176712 32321924

[17] Fornaciai M, Park J. Serial dependence generalizes across different stimulus formats, but not different sensory modalities. Vision Res. 2019;160:108–15. 10.1016/j.visres.2019.04.011. 31078663

[18] Fornaciai M, Park J. The effect of abstract representation and response feedback on serial dependence in numerosity perception. Atten Percept Psychophys. 2022;84(5):1651–65. 10.3758/s13414-022-02518-y. 35610413

[19] Walsh V. A theory of magnitude: common cortical metrics of time, space and quantity. Trends Cogn Sci. 2003;7(11):483–8. 10.1016/j.tics.2003.09.002. 14585444

[20] Bueti D, Walsh V. The parietal cortex and the representation of time, space, number and other magnitudes. Philos Trans R Soc Lond B Biol Sci. 2009;364(1525):1831–40. 10.1098/rstb.2009.0028. PMC2685826 19487186

[21] Javadi AH, Aichelburg C. When time and numerosity interfere: the longer the more, and the more the longer. PLoS ONE. 2012;7(7):e41496. 10.1371/journal.pone.0041496. PMC3401119 22911801

[22] Togoli I, Fornaciai M, Bueti D. The specious interaction of time and numerosity perception. Proc R Soc B Biol Sci. 2021;288(1959):20211577. 10.1098/rspb.2021.1577. PMC8456131 34547911

[23] Togoli I, Bueti D, Fornaciai M. The nature of magnitude integration: contextual interference versus active magnitude binding. J Vis. 2022;22(11):11. 10.1167/jov.22.11.11. PMC9587468 36259675

[24] Lambrechts A, Walsh V, van Wassenhove V. Evidence accumulation in the magnitude system. PLoS ONE. 2013;8(12):e82122. 10.1371/journal.pone.0082122. PMC3855382 24339998

[25] Casasanto D, Boroditsky L. Time in the mind: using space to think about time. Cognition. 2008;106(2):579–93. 10.1016/j.cognition.2007.03.004. 17509553

[26] Tsouli A, van der Smagt MJ, Dumoulin SO, te Pas SF. Distinct temporal mechanisms modulate numerosity perception. J Vis. 2019;19(6):19. 10.1167/19.6.19. 31238340

[27] Tsouli A, Dumoulin SO, te Pas SF, van der Smagt MJ. Adaptation reveals unbalanced interaction between numerosity and time. Cortex. 2019;114:5–16. 10.1016/j.cortex.2018.02.013. 29571959

[28] Fornaciai M, Togoli I, Arrighi R. Motion-induced compression of perceived numerosity. Sci Rep. 2018. 10.1038/s41598-018-25244-8. PMC5934405 29725026

[29] Fornaciai M, Arrighi R, Burr DC. Adaptation-induced compression of event time occurs only for translational motion. Sci Rep. 2016;6. 10.1038/srep23341. PMC4802346 27003445

[30] Togoli I, Fedele M, Fornaciai M, Bueti D. Serial dependence in time and numerosity perception is dimension-specific. J Vis. 2021;21(5):6. 10.1167/jov.21.5.6. PMC8107483 33956059

[31] Togoli I, Collignon O, Bueti D, Fornaciai M. The mechanisms and neural signature of time-averaged numerosity perception. J Cogn Neurosci. 2024;37(2):1–17. 10.1162/jocn_a_02263. 39436233

[32] Fornaciai M, Togoli I, Bueti D. Perceptual history biases are predicted by early visual-evoked activity. J Neurosci. 2023;43(21):3860–75. 10.1523/JNEUROSCI.1451-22.2023. PMC10218026 37085319

[33] Restle F. Speed of adding and comparing numbers. J Exp Psychol. 1970;83(2, Pt.1):274–8. 10.1037/h0028573.

[34] Dehaene S, Bossini S, Giraux P. The mental representation of parity and number magnitude. J Exp Psychol Gen. 1993;122(3):371–96. 10.1037/0096-3445.122.3.371.

[35] Fias W. The importance of magnitude information in numerical processing: evidence from the SNARC effect. Math Cogn. 1996;2(1):95–110. 10.1080/135467996387552.

[36] Gevers W, Lammertyn J, Notebaert W, Verguts T, Fias W. Automatic response activation of implicit spatial information: evidence from the SNARC effect. Acta Psychol (Amst). 2006;122(3):221–33. 10.1016/j.actpsy.2005.11.004. 16423320

[37] Eccher E, Josserand M, Caparos S, Boissin E, Buiatti M, Piazza M, et al. A left-to-right bias in number-space mapping across ages and cultures. Nat Commun. 2025;16(1):495. 10.1038/s41467-024-55685-x. PMC11724025 39794321

[38] Shaki S, Petrusic WM, Leth-Steensen C. SNARC effects with numerical and non-numerical symbolic comparative judgments: instructional and cultural dependencies. J Exp Psychol Hum Percept Perform. 2012;38(2):515–30. 10.1037/a0026729. 22288694

[39] Rugani R, Vallortigara G, Priftis K, Regolin L. Number-space mapping in the newborn chick resembles humans’ mental number line. Science (1979). 2015;347(6221):534–6. 10.1126/science.aaa1379. 25635096

[40] Scozia G, Pinto M, Lozito S, Lasaponara S, Binetti N, Pazzaglia M, et al. Space is a late heuristic of elapsing time: new evidence from the STEARC effect. Cortex. 2023;164:21–32. 10.1016/j.cortex.2023.03.009. 37148825

[41] Sadil P, Cowell RA, Huber DE. The push–pull of serial dependence effects: attraction to the prior response and repulsion from the prior stimulus. Psychon Bull Rev. 2024;31(1):259–73. 10.3758/s13423-023-02320-3. PMC11488665 37566217

[42] Cicchini GM, Mikellidou K, Burr DC. The functional role of serial dependence. Proc R Soc B Biol Sci. 2018;285(1890):20181722. 10.1098/rspb.2018.1722. PMC6235035 30381379

[43] Burgio I, Moscoso PAM, Anobile G, Castaldi E. Stronger reliance on visual perceptual history in individuals with higher math anxiety. BMC Biol. 2025;23(1):305. 10.1186/s12915-025-02417-2. PMC12522993 41088111

[44] Anobile G, Burr DC, Gasperini F, Cicchini GM. Near optimal encoding of numerosity in typical and dyscalculic development. Cortex. 2019;120:498–508. 10.1016/j.cortex.2019.07.009. 31520845

[45] Kanai R, Paffen CLE, Hogendoorn H, Verstraten FAJ. Time dilation in dynamic visual display. J Vis. 2006;6(12):8. 10.1167/6.12.8. 17209745

[46] Fornaciai M, Park J. Spatiotemporal feature integration shapes approximate numerical processing. J Vis. 2017. 10.1167/17.13.6. 29114806

[47] Cai ZG, Connell L. Space–time interdependence: evidence against asymmetric mapping between time and space. Cognition. 2015;136:268–81. 10.1016/j.cognition.2014.11.039. 25506776

[48] Temple E, Posner MI. Brain mechanisms of quantity are similar in 5-year-old children and adults. Proc Natl Acad Sci U S A. 1998;95(13):7836–41. 10.1073/pnas.95.13.7836. PMC22775 9636237

[49] Libertus ME, Woldorff MG, Brannon EM. Electrophysiological evidence for notation independence in numerical processing. Behav Brain Funct. 2007;3(1):1. 10.1186/1744-9081-3-1. PMC1781950 17214890

[50] Fornaciai M, Park J. Early numerosity encoding in visual cortex is not sufficient for the representation of numerical magnitude. J Cogn Neurosci. 2018;30(12):1788–802. 10.1162/jocn_a_01320. 30063175

[51] Fornaciai M, Park J. Distinct neural signatures for very small and very large numerosities. Front Hum Neurosci. 2017;11. 10.3389/fnhum.2017.00021. PMC5282473 28197086

[52] Fornaciai M, Brannon EM, Woldorff MG, Park J. Numerosity processing in early visual cortex. Neuroimage. 2017;157:429–38. 10.1016/j.neuroimage.2017.05.069. PMC6697050 28583882

[53] Whitney D, Yamanashi LA. Ensemble perception. Annu Rev Psychol. 2018;69(1):105–29. 10.1146/annurev-psych-010416-044232. 28892638

[54] McDermott JH, Schemitsch M, Simoncelli EP. Summary statistics in auditory perception. Nat Neurosci. 2013;16(4):493–8. 10.1038/nn.3347. PMC4143328 23434915

[55] Hubert-Wallander B, Boynton GM. Not all summary statistics are made equal: evidence from extracting summaries across time. J Vis. 2015;15(4):5. 10.1167/15.4.5. PMC4463776 26053144

[56] Ceylan G, Herzog MH, Pascucci D. Serial dependence does not originate from low-level visual processing. Cognition. 2021;212:104709. 10.1016/j.cognition.2021.104709. 33838523

[57] Cicchini GM, Benedetto A, Burr DC. Perceptual history propagates down to early levels of sensory analysis. Curr Biol. 2021;31(6):1245-1250.e2. 10.1016/j.cub.2020.12.004. PMC7987721 33373639

[58] Fornaciai M, Park J. Disentangling feedforward versus feedback processing in numerosity representation. Cortex. 2021;135:255–67. 10.1016/j.cortex.2020.11.013. 33412370

[59] Fornaciai M, Park J. Attractive serial dependence in the absence of an explicit task. Psychol Sci. 2018;29(3):437–46. 10.1177/0956797617737385. 29381415

[60] Fornaciai M, Park J. Spontaneous repulsive adaptation in the absence of attractive serial dependence. J Vis. 2019;19(5):21. 10.1167/19.5.21. 31112999

[61] Petrizzo I, Pellegrino M, Anobile G, Doricchi F, Arrighi R. Top-down determinants of the numerosity–time interaction. Sci Rep. 2023;13(1):21098. 10.1038/s41598-023-47507-9. PMC10689472 38036544

[62] Cui M, Peng C, Huang M, Chen Y. Electrophysiological evidence for a common magnitude representation of spatiotemporal information in working memory. Cereb Cortex. 2022. 10.1093/cercor/bhab466. 35024791

[63] Harvey BM, Fracasso A, Petridou N, Dumoulin SO. Topographic representations of object size and relationships with numerosity reveal generalized quantity processing in human parietal cortex. Proc Natl Acad Sci U S A. 2015;112(44):13525–30. 10.1073/pnas.1515414112. PMC4640722 26483452

[64] Fortunato G, Togoli I, Bueti D. The more numerous the longer: how the integration between numerosity and time leads to a common neural response. Proceedings of the Royal Society B: Biol Sci. 2023;290(1998). 10.1098/rspb.2023.0260. PMC10170217 37161323

[65] Hendrikx E, Paul JM, van Ackooij M, van der Stoep N, Harvey BM. Cortical quantity representations of visual numerosity and timing overlap increasingly into superior cortices but remain distinct. Neuroimage. 2024;286:120515. 10.1016/j.neuroimage.2024.120515. 38216105

[66] Manassi M, Whitney D. Illusion of visual stability through active perceptual serial dependence. Sci Adv. 2022;8(2). 10.1126/sciadv.abk2480. PMC11580026 35020432

[67] Murai Y, Whitney D. Serial dependence revealed in history-dependent perceptual templates. Curr Biol. 2021;31(14):3185-3191.e3. 10.1016/j.cub.2021.05.006. PMC8319107 34087105

[68] Manassi M, Whitney D. Continuity fields enhance visual perception through positive serial dependence. Nature Reviews Psychology. 2024;3(5):352–66. 10.1038/s44159-024-00297-x. PMC12890194 41675977

[69] Togoli I, Collignon O, Bueti D, Fornaciai M. Magnitude processing and integration entail perceptual processes independent from the task. Imaging Neuroscience. 2025;24:3. 10.1162/imag_a_00485. PMC12319895 40800801

[70] Samaha J, Switzky M, Postle BR. Confidence boosts serial dependence in orientation estimation. J Vis. 2019;19(4):25. 10.1167/19.4.25. PMC6690400 31009526

[71] Kleiner M, Brainard D, Pelli D. What’s new in Psychtoolbox-3? Perception 36 ECVP Abstract Supplement. 2007.

[72] Pelli DG. The VideoToolbox software for visual psychophysics: transforming numbers into movies. Spat Vis. 1997;10:437–42. 9176953

