# Supplementary material for: Cross-dimension serial dependence effects between numerosity and duration
Source: BMC Biol. 2026 Jul 27;24:230. doi: 10.1186/s12915-026-02701-9 (PMC13749872; doi:10.1186/s12915-026-02701-9)
Supplement: Supplementary file 1 [file 12915_2026_2701_MOESM1_ESM.pdf]

ADDITIONAL FILE 1

Cross-dimension serial dependence effects between numerosity and duration

Fornaciai M., Togoli I., Collignon, O.

1. Analysis of response times in the numerosity task

Differently from the duration task, where the response time (RT) corresponds to the judgment of the stimulus, RTs in the numerosity task represent an additional factor that should be taken into account. One potential confound that we need to address is indeed the association between space and magnitude, which may result in a facilitation or an interference with the judgment. For example, according the spatial-numerical association of response codes (SNARC) effect, the compatibility of response codes (e.g., the side of space in which the response takes place) with the magnitude can affect the speed of response (1–7). Typically, low magnitudes are associated with the left space, and high magnitudes to the right space. A facilitation (faster RTs) thus occurs when the response code for low magnitudes is placed on the left, and the one for high magnitudes on the right (e.g., buttons on the left and right side of a keyboard, respectively), and an interference (slower RTs) when the response codes are inconsistent with the magnitude spatial mapping. This effect has been demonstrated not only with numbers and numerosities, but also with duration (8). Thus, it is important to assess whether the compatibility of magnitudes and response codes could have affected the judgement, to rule out a role of such associations in the observed magnitude integration effects.

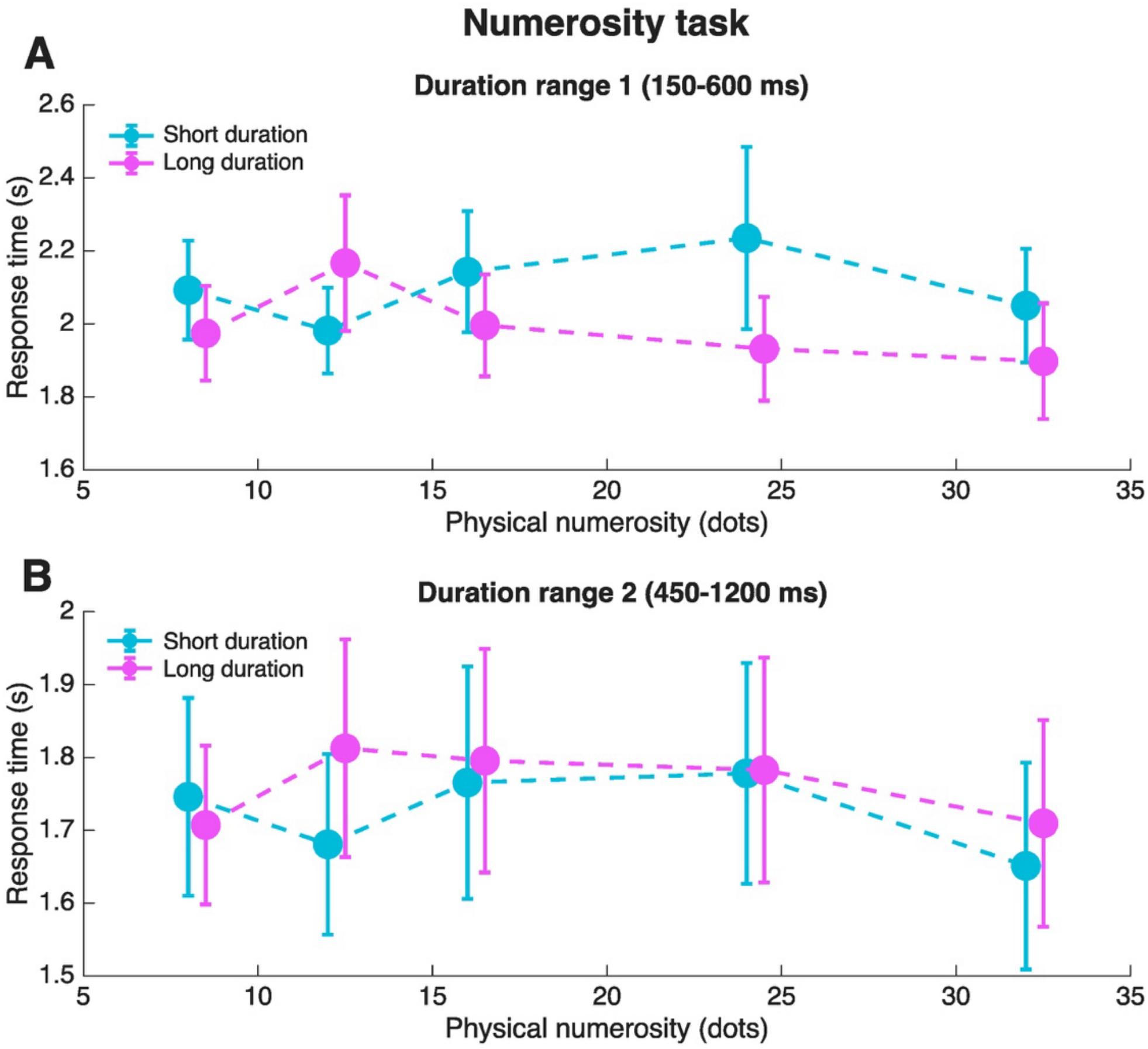

FIGURE S1. Response times in the numerosity task as a function of the different numerosities and the task-irrelevant durations. *(A) Average response times with the duration range 1 (blocks 1, 3, 5). (B) Average response times with the duration range 2 (blocks 2, 4). Error bars are SEM.*

To address this point, we computed the average RTs in the numerosity task as a function of the different levels of numerosity being estimated, and whether the numerosity was associated with a short (150-200 ms) or long (450-600 ms) duration. In terms of effects, if the response compatibility affected the judgment of numerosity, we would expect the combination of numerosity and duration of the stimuli to modulate the response time. For example, when judging a high numerosity (i.e., response occurring on the right part of the slider), a long duration should facilitate the response, while a short duration should hinder it. Conversely, when judging a low numerosity (i.e., response occurring on the left part of the slider), the opposite should occur. Fig. S1 above shows the average response

times, separately for the duration range 1 (150-600 ms; blocks 1, 3, 5) and range 2 (450-1200 ms; blocks 2, 4). To assess whether the RTs are significantly modulated by the compatibility of duration and response codes, we used two linear mixed effect models (LME) on RTs, including numerosity (8, 12, 16, 24, 32 dots) and duration (short vs. long) as fixed effects, and random effects representing the subjects as well as the numerosity and duration themselves, according to the following formula:

RTs ~ Numerosity x Duration + (1|subj) + (1|Numerosity) + (1|Duration)

With the duration range 1 (Fig. S1A), the average RTs showed a distribution at around 2 s, with however little difference between different numerosity levels and different durations. The LME test ($R^2 = 0.61$) indeed did not show any significant main effect of numerosity ($b = 0.07$, $t = 1.01$, $p = 0.31$) and duration ($b = 0.06$, $t = 0.39$, $p = 0.69$), and no interaction between the two factors ($b = -0.05$, $t = -1.22$, $p = 0.22$).

With the duration range 2 (Fig. S1B), we observed overall slightly lower RTs, distributed at around 1.75 s. Also in this case, the LME test ($R^2 = 0.89$) did not show any significant main effect of numerosity ($b = -0.02$, $t = -0.45$, $p = 0.65$) or duration ($b = 0.02$, $t = 0.23$, $p = 0.81$), and no interaction between the two factors ($b = 0.01$, $t = 0.31$, $p = 0.76$).

These results thus show that the compatibility between the interfering magnitude and the response code of the task-relevant magnitude did not influence the response speed, which would be the main type of influence expected from a response compatibility effect. Additionally, they show that the response times in the numerosity task, in general, did not significantly vary neither as a function of numerosity, nor duration. The RTs and the response compatibility are therefore unlikely to have contributed to the observed magnitude integration effect.

2. **Analysis of the relationship between serial dependence and magnitude integration effects with perceptual precision**

The serial dependence effect has often been linked with the variability of perceptual precision in the task, both within (9) and across participants (10–12). Typically, the serial dependence effect, defined as the influence that the past percept has on the current percept, has been associated with increasing perceptual thresholds. That is, the lower the precision (e.g., higher Weber's fraction), the stronger the effect.

To address whether a relationship with perceptual precision might represent another common factor between the serial dependence and magnitude integration effect, we assessed the correlation of these two biases with the precision in the tasks, in terms of Weber's fraction (WF). More specifically, we used the serial dependence effect index and the magnitude integration effect index in percentage (see Methods in the main manuscript), and assessed the correlation with the Weber' fraction as a measure of precision. This was done separately for the blocks where we used the first and the second range.

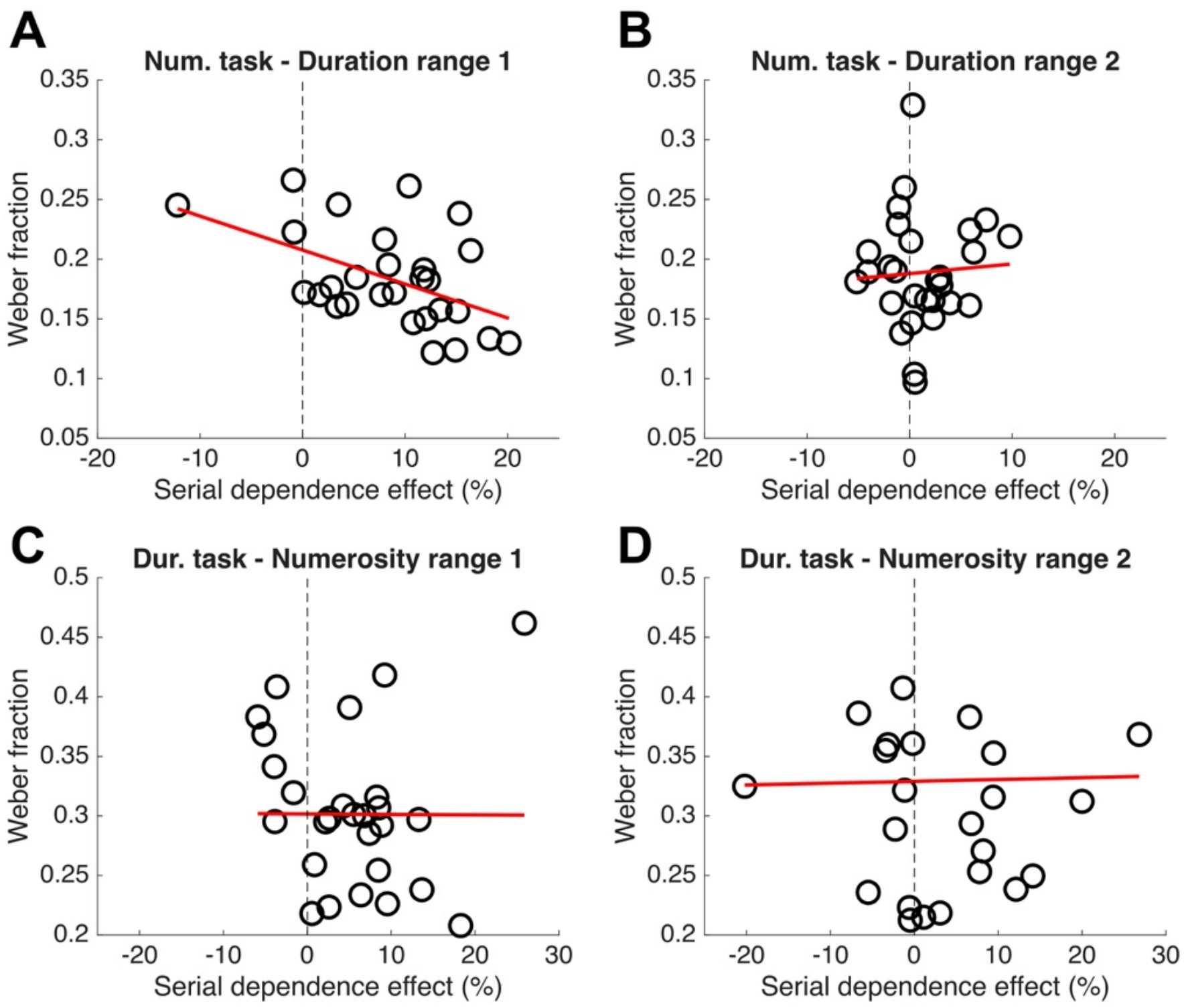

FIGURE S2. Relationship between the serial dependence effect and the precision in the task. *(A) Correlation between the serial dependence effect and Weber's fraction (WF) in the numerosity task, with the duration range 1. (B) Correlation between the serial dependence effect and WF in the numerosity task, with the duration range 2. (C) Correlation between the serial dependence effect and WF in the duration task, with the numerosity range 1. (D) Correlation between the serial dependence effect and WF in the duration task, with the numerosity range 2. The red lines plotted in each panel represent the regression line based on the data displayed.*

Regarding the serial dependence effect (Fig. S2), in the numerosity task we observed a significant negative correlation between serial dependence and Weber's fraction (WF) in the blocks in which we used the duration range 1 ($r = -0.49$, $p = 0.008$; Fig. S2A). This correlation shows that in this case stronger serial dependence effects are associated with higher precision (i.e., lower WF). However, no significant correlation was observed in the numerosity task when using the duration range 2 ($r = 0.06$, $p = 0.76$; Fig. S2B), possibly due to the very small effects occurring in this case, as well as in the duration task ($r = -0.004$, $p = 0.98$ and $r = 0.01$, $p = 0.94$, respectively for the numerosity range 1 and 2; Fig. S2C-D). These results suggest that a relationship between serial dependence and precision emerges mostly in the numerosity task, and only when the effect is strong enough to significantly change the perception of the current stimulus. In the duration task, despite the strong and significant effects measured with the numerosity range, no consistent relationship emerged. These results might suggest a partial dissociation between the effects occurring in different tasks, with the mechanisms of serial dependence including a task-specific component.

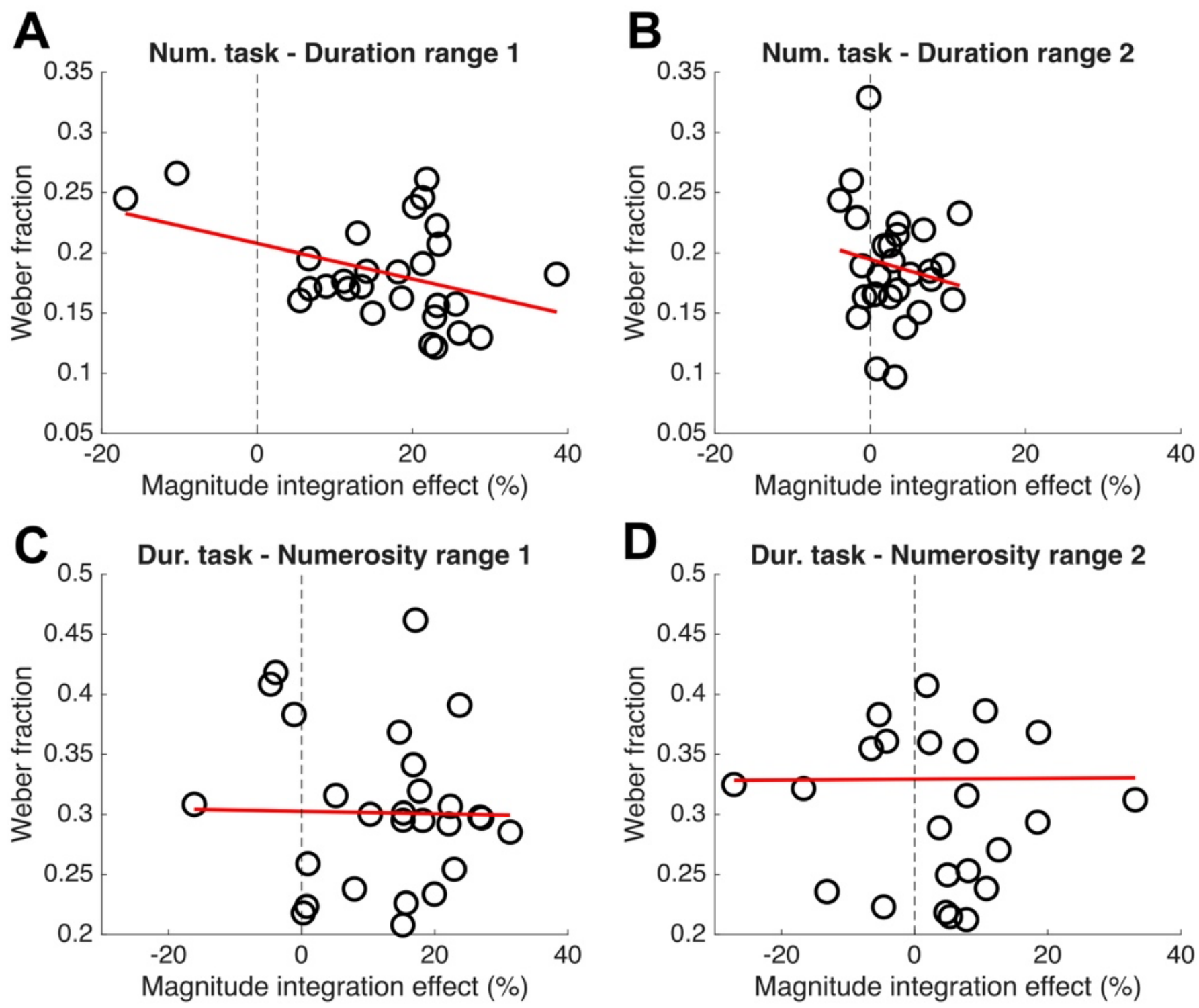

FIGURE S3. Relationship between the magnitude integration effect and the precision in the task. *(A) Correlation between magnitude integration and Weber's fraction (WF) in the numerosity task, with the duration range 1. (B) Correlation between magnitude integration and WF in the numerosity task, with the duration range 2. (C) Correlation between magnitude integration and WF in the duration task, with the numerosity range 1. (D) Correlation between magnitude integration and WF in the duration task, with the numerosity range 2. The red lines plotted in each panel represent the regression line based on the data displayed.*

Regarding the magnitude integration effect, we similarly observed a significant correlation between the strength of the effect and the Weber's fraction in the numerosity task with the duration range 1 ($r = -0.41$, $p = 0.032$; Fig. S3A). Again, similarly to the serial dependence effect, we did not find any significant correlation in the numerosity task with the duration range 2 ($r = -0.16$, $p = 0.41$; Fig. S3B), and in the duration task ($r = -0.02$, $p = 0.93$, Fig. S3C; $r = 0.004$, $p = 0.98$; Fig. S3D).

The similar correlation of the serial dependence effect and magnitude integration effect with the perceptual precision presents an interesting additional link between the two effects. However, this is limited to the numerosity task, and to the case where we used the first duration range. Overall, although the two biases correlate with each other (see main manuscript), this shows that the underlying mechanisms of these two effects have at least a task-specific component. We cannot however exclude that the presence or absence of a correlation in these tests might be related to the specific tasks used rather than the mechanisms of serial dependence and magnitude integration, since the numerosity and duration task were very different. This interpretation thus remains speculative, and the different relationships between effects and perceptual precision remain a possible interesting topic for future investigations.
